# Functional Investigation of the Plant-Specific Long Coiled-Coil Proteins PAMP-INDUCED COILED-COIL (PICC) and PICC-LIKE (PICL) in *Arabidopsis thaliana*

**DOI:** 10.1371/journal.pone.0057283

**Published:** 2013-02-25

**Authors:** Sowmya Venkatakrishnan, David Mackey, Iris Meier

**Affiliations:** 1 Department of Molecular Genetics, The Ohio State University, Columbus, Ohio, United States of America; 2 Department of Horticulture and Crop Science, The Ohio State University, Columbus, Ohio, United States of America; Iowa State University, United States of America

## Abstract

We have identified and characterized two Arabidopsis long coiled-coil proteins PAMP-INDUCED COILED-COIL (PICC) and PICC-LIKE (PICL). PICC (147 kDa) and PICL (87 kDa) are paralogs that consist predominantly of a long coiled-coil domain (expanded in PICC), with a predicted transmembrane domain at the immediate C-terminus. Orthologs of PICC and PICL were found exclusively in vascular plants. PICC and PICL GFP fusion proteins are anchored to the cytoplasmic surface of the endoplasmic reticulum (ER) membrane by a C-terminal transmembrane domain and a short tail domain, via a tail-anchoring mechanism. T-DNA-insertion mutants of *PICC* and *PICL* as well as the double mutant show an increased sensitivity to the plant abiotic stress hormone abscisic acid (ABA) in a post-germination growth response. *PICC,* but not *PICL* gene expression is induced by the bacterial pathogen-associated molecular pattern (PAMP) flg22. T-DNA insertion alleles of *PICC,* but not *PICL,* show increased susceptibility to the non-virulent strain *P. syringae* pv. *tomato* DC3000 *hrcC*, but not to the virulent strain *P. syringae* pv. *tomato* DC3000. This suggests that *PICC* mutants are compromised in PAMP-triggered immunity (PTI). The data presented here provide first evidence for the involvement of a plant long coiled-coil protein in a plant defense response.

## Introduction

Plants have a highly sensitive system for perceiving pathogen attack, which consists of multiple layers. The first layer involves the recognition of pathogen-associated molecular patterns (PAMPs, also referred to as microbe-associated molecular patterns or MAMPs), which are conserved and abundant molecular components present across a broad range of microorganisms and essential for the microbial life style [1]. Bacterial flagellin, elongation factor-Tu (EF-Tu), lipopolysaccharides, and fungal chitin are some examples of PAMPs that are recognized by plants [1], [2], [3], [4]. Pattern recognition receptors (PRRs), present on the surface of plant cells, detect the presence of PAMPs and activate the first layer of defense called PAMP-triggered immunity (PTI).

Flagellin, the building block of the bacterial flagellar filament, contains the best-characterized PAMP [2]. A conserved N-terminal 22 amino acid epitope of flagellin, flg22, is sufficient to induce PTI in most plant species [2]. flg22 is recognized in Arabidopsis by the PRR FLAGELLIN SENSITIVE2 (FLS2) [5]. PTI initiation by FLS2 and other PRRs results in signaling events and defense responses such as generation of reactive oxygen species (ROS), accumulation of the plant hormones ethylene and salicylic acid (SA), activation of mitogen-activated protein kinase (MAPK) signaling cascades, global transcriptional reprogramming, secretion of anti-microbial compounds, and callose deposition at the cell wall. PTI contributes to resistance against potentially pathogenic microbes.

*P. syringae* pv. *tomato* DC3000 (*Pst*DC3000), which is virulent on tomato and Arabidopsis, counteracts PTI by using type III secretion to deliver defense suppressing effector proteins into the host plant cytoplasm [6], [7]. The *Pst*DC3000 *hrcC* mutant, which is defective in type III secretion, fails to suppress PTI and consequently grows poorly in plants [8], [9], [10]. In response to PTI-suppressing effectors, intracellular resistance (R) proteins recognize individual effectors and elicit effector-triggered immunity (ETI), often a more heightened and sustained immune response [11]. The predominate class of R proteins involved in effector recognition is the nucleotide binding site-leucine rich repeat (NBS-LRR) type proteins, of which coiled-coil-NBS-LRR (CC-NBS-LRR) proteins are a subclass [11], [12].

Coiled-coils are protein domains that consist of two or more α helices that wrap around each other to form a super-coil [13], [14]. They are ubiquitous protein motifs predicted to be present in ∼10% of all proteins in eukaryotes [14]. Coiled-coil proteins can roughly be divided into two classes, containing “short” or “long” coiled-coil domains [15]. Short coiled-coil domains of six or seven heptad repeats mediate homo- and heterodimerization of the basic region leucine zipper (bZIP) family of transcription factors [16]. Tetrameric short coiled-coil structures are involved in *N-ethylmaleimide-sensitive* factor attachment protein receptor (SNARE)-mediated fusions of vesicles to their destination compartments [17], [18].

Long coiled-coil domains, composed of several hundred amino acids are found in functionally diverse proteins [15]. These include, among others, the intermediate filament proteins, proteins involved in organelle architecture and in nuclear organization, and the cytoskeletal motor proteins [14], [19], [20], [21], [22]. Golgins are a family of predominantly coiled-coil proteins that localize to the cytoplasmic surface of the Golgi apparatus, maintain Golgi architecture, and regulate vesicle trafficking through their coiled-coil domains [23], [24].

Little is known about the function of long coiled-coil proteins in plants. In Arabidopsis, 286 proteins are predicted to have long coiled-coil domains [15], but few have been functionally investigated [25], [26], [27]. However, even with a small sample size, a significant number of investigated long coiled-coil proteins were found to be plant specific. These include in the cytoplasm the WPP interacting proteins (WIPs), plant-specific nuclear anchors of Ran GTPase Activating Protein 1 (RanGAP1) [28] and COP1 interacting protein (CIP1) a cytoskeleton-associated interactor of constitutive photomorphogenic protein (COP1) [29], [30]. In plastids, MAR binding filament-like protein 1 (MFP1) is associated with nucleoids and thylakoid membranes [31], Chloroplast Unusual Positioning 1 (CHUP1) anchors the chloroplast to the plasma membrane, and weak chloroplast movement under blue light 1 (WEB1) and plastid movement impaired 2 (PMI2) are involved in chloroplast movement [32], [33]. PEROXISOMAL AND MITOCHONDRIAL DIVISION FACTOR1 (PMD1) and its homolog PMD2 play non-redundant functions in organelle morphogenesis and proliferation [34]. None of these proteins has homologs outside the plant lineage.

The discovery of plant-specific long coiled-coil proteins and their functional characterization suggests that some of the currently still uncharacterized proteins might be involved in plant specific processes such as photosynthesis, plant-specific aspects of cytokinesis or plant defense mechanisms. In this study, we report the characterization of a family of two novel plant-specific long coiled-coil proteins, PAMP-INDUCED COILED COIL (PICC) and PICC-like (PICL). They are endoplasmic reticulum (ER)-localized tail-anchored proteins with the coiled-coil domains facing the cytoplasm. *PICC* gene expression is rapidly induced by PAMPs and a *picc* null mutant shows compromised resistance against *hrcC* bacteria. While *PICL* is not induced by PAMPs, and appears to play no role in PTI, both *picc* and *picl* T-DNA insertion mutants show a modulated post-germination ABA response.

## Materials and Methods

### Plant Materials and Growth Conditions

All *Arabidopsis thaliana* plants used in this study were in the Col-0 background. T-DNA insertion alleles *picc-1* (SALK_58801), *picc-2* (SALK_139837) and *picl-1* (SALK_56040) were obtained from the Arabidopsis Biological Resource Center (ABRC, Columbus, OH, USA). The homozygous mutant lines were identified by PCR of genomic DNA using the primers listed in Table S1. To grow Arabidopsis seedlings used for quantitative PCR assays, seeds were sterilized in 40% v/v hypochlorite, washed six times with sterile water and germinated in 6-well microtiter dishes (∼15–20 seeds per well) containing liquid Murashige and Skoog (MS) media (1x MS basal salts (Caisson, Logan, UT, USA)), 1% sucrose, 0.5 gl^−1^ MES, 1x Gamborg’s vitamins (Sigma, St. Louis, MO, USA), pH 5.7) and sealed with parafilm. The seedlings were grown in a plant growth chamber under long day conditions (16 h light/8 h dark) at 22°C. Arabidopsis plants used for quantitative RT-PCR, ROS measurements and bacterial growth curve assays were grown in soil at 22°C (light)/18°C (dark) under short day conditions (8 h light/16 h dark). Arabidopsis plants used for all other experiments were grown in soil at 22°C under standard long day conditions (16 h light/8 h dark). *Nicotiana benthamiana* plants were grown in soil under standard long-day conditions at 24°C.

### Constructs and Cloning

For localization assays, the PICC and PICL ORF were amplified using the Thermoscript RT-PCR system (Invitrogen, Carlsbad, CA, USA) and ProSTAR HF Single Tube RT-PCR System (Agilent, Santa Clara, CA, USA), respectively. The PICC and PICL cDNAs were then cloned into pDONR221 and pENTR/D-TOPO Gateway entry vectors (Invitrogen), respectively. PICCΔTDF and PICLΔTDF were amplified from PICC and PICL cDNA using Phusion polymerase (New England Biologicals (NEB), Ipswich, MA, USA) and cloned into pDONR221 entry vector. TDF^PICC^ and TDF^PICL^ were also amplified from PICC and PICL cDNAs using Phusion polymerase and cloned into the pDONR221 entry vector. PICC, PICL and their deletion variants were moved from the entry vectors into the Gateway destination vector pK7WGF2 (Invitrogen) by LR recombination. For GUS assays, 2.0 kb PICC promoter (*pPICC*) and 1.0 kb PICL promoter (*pPICL*) were amplified from the WT Col-0 genomic DNA and cloned into pDONR221 and pENTR/D-TOPO entry vectors, respectively. *pPICC* and *pPICL* were moved into destination vectors pGWB1 and pMDC162 respectively. All the clones in the destination vectors were introduced into *Agrobacterium tumefaciens* (Agrobacterium) strain GV3101. For split-ubiquitin membrane yeast two-hybrid, PICC and PICL were cloned into Cub and Nub vectors, pBT3N and pPR3N (Dual Systems Biotech, Switzerland) using the In-Fusion cloning system (Clontech, Mountainview, CA). The ER marker HDEL-mCherry (CD3-959) was obtained from the ABRC.

GFP-CXN and CXN-PAGFP used as controls for protease protection assay were kindly donated by Chris Hawes, Oxford Brookes University. The primers used for cloning are listed in Table S2. Sequences of all clones in the entry vectors and in the split-ubiquitin membrane yeast two-hybrid vectors were verified by sequencing at the Plant-Microbe Genomics Facility (PMGF, The Ohio State University, Columbus, USA).

### Generation and Selection of Arabidopsis Transgenic Lines

Transgenic lines GFP-PICC, GFP-PICL, GFP-PICCΔTDF, GFP-PICLΔTDF, GFP-TDF^PICC^, GFP-TDF^PICL^, pPICC::GUS, pPICL::GUS, were generated by transforming Arabidopsis wild type Col-0 plants with Agrobacterium strain GV3101 carrying individual plasmids, by the floral dipping method [35]. Transgenic T1 progeny were selected on agar plates containing MS medium (1x MS basal salts (Caisson), 1% sucrose, 0.5 gl^−1^ MES, 1x Gamborg’s vitamins (Sigma) and 0.8% agar) with 50 µgml^−1^ kanamycin or 50 µgml^−1^ hygromycin or both. For localization analysis, T1 progeny carrying GFP-fusion genes of interest were analyzed by confocal laser scanning microscopy. For promoter analysis, at least 5 independent T2 lines were selected and subjected to staining for GUS expression.

For ER morphology analysis, transgenic HDEL-mCherry plants were generated by transforming WT and *picc-1;picl-1* with Agrobacterium strain GV3101 carrying HDEL-mCherry driven by Cauliflower mosaic virus 35S promoter. Transgenic T1 progeny were selected on agar plates containing MS medium (1x MS basal salts (Caisson), 1% sucrose, 0.5 gl^−1^ MES, 1x Gamborg’s vitamins (Sigma) and 0.8% agar) with 50 µgml^−1^ kanamycin. T1 progeny carrying HDEL-mCherry were analyzed by confocal laser scanning microscopy.

### Transient Expression of Proteins in *N. benthamiana* Leaf Epidermal Cells

To transiently express GFP-fusion proteins of interest, Agrobacterium cultures containing different plasmids were coinfiltrated with Agrobacterium cultures carrying HDEL-mCherry into leaves of 3- to 4-week-old *N. benthamiana* plants as described previously [36]. Agrobacterium cells carrying plasmids of interest were resuspended in a solution containing 10 mM MgCl_2_, 10 mM MES and 100 µM acetosyringone. The O.D. of each Agrobacterium culture was adjusted to A_600_ = 0.2, and for co-infiltration, cultures were mixed in a ratio of 1∶1 and syringe infiltrated into *N. benthamiana* leaves. The GFP and mCherry expression patterns were analyzed by confocal microscopy 48 h after infiltration.

### Confocal Laser Scanning Microscopy

All images were acquired using a confocal laser scanning microscope (Nikon D-ECLIPSE C1 90i). GFP fluorescence was observed using an excitation wavelength of 488 nm and emission wavelength of 515/530 nm. mCherry was observed using an excitation wavelength of 543 nm and emission wavelength of 560/615 nm.

### Protease Protection Assay

Protease protection assays were performed as described [37]. Agrobacterium infiltrated Arabidopsis leaf sectors (∼100 mg) expressing the relevant proteins at ∼48 h after infiltration were ground to homogeneity in a mortar and pestle (chilled at 4°C) in 500 µl ice-cold extraction buffer (100 mM Tris-HCl pH 7.6, 10 mM KCl, 1 mM EDTA and 12% w/w sucrose). The homogenate was centrifuged at 5000 rpm for 5 min at 4°C to sediment the debris and the supernatant was centrifuged at 20,000 g for 20 min at 4°C. The supernatant was layered onto 17% sucrose buffer (in water) and centrifuged at 100,000 g for 1 h at 4°C. The sediment obtained was resuspended in ice-cold extraction buffer. 75 µl of each sample was added to each of four tubes each containing 1 mM CaCl_2_+ PK buffer (50 mM Tris-HCl pH 8.0, 1 mM CaCl_2_) with or without proteinase K (200 µg ml^−1^, NEB) or 1 mM CaCl_2_+1% Triton X-100+ PK buffer (50 mM Tris-HCl pH 8.0, 1 mM CaCl_2_) with or without proteinase K (200 µg ml^−1^) and incubated at 25°C for 30 min. To terminate the reaction, 1 µl of protease inhibitor cocktail (Sigma-Aldrich) was added to each tube and incubated at 25°C for 10 min. 3x SDS protein loading buffer (150 mM Tris-HCl pH 6.8, 6% SDS, 300 mM DTT, 30% glycerol, 0.3% bromophenol blue) was added and samples were boiled for 5 min before subjecting to SDS-PAGE on a 15% SDS-polyacrylamide gel. Immunoblot analysis with anti-GFP antibody (Invitrogen) was performed as described below.

### ß-Glucuronidase Staining

Arabidopsis tissues were prefixed in ice-cold 90% acetone for 1 h and then immersed in staining solution (50 mM sodium phosphate buffer pH 7.0, 10 mM EDTA, 2 mM potassium ferricyanide, 2 mM potassium ferrocyanide, 0.1% Triton X-100 and 2 mM 5-bromo-4-chloro-3-indolyl-β-D-glucuronic acid) at 37°C for 16 h. Tissues were rinsed three times in 90% ethanol and stored in 70% ethanol at room temperature (RT) until examination. Micrographs were taken using a Nikon Digital Sight DS–5M camera attached to a Nikon SMZ800 dissecting microscope or a Nikon Eclipse 80i compound microscope.

### Membrane Yeast Two Hybrid (Split Ubiquitin) Assay

Yeast competent cell preparation and transformation was carried out as described [38]. Transformants were selected on yeast dropout media (SD -leucine -tryptophan). Three colonies were picked from each transformation to perform β-galactosidase assays. To quantify β-galactosidase activity, yeast cells were grown at 28°C to A_600_ = 1 and chilled on ice for 15 min. 2 ml of the culture was then centrifuged and the sediment was frozen in liquid nitrogen and resuspended in resuspension buffer consisting of 665 µl “H” buffer (150 mM NaCl, 100 mM HEPES, 2 mM MgCl_2_ and 1% (w/v) BSA, pH 7.0), 55 µl chloroform, 55 µl 0.1% (w/v) SDS and 125 µl 0.4% (w/v) 2-Nitrophenyl β-D-galactopyranoside (ONPG)). The suspension was incubated at 30°C until visible yellow color developed. The reaction was stopped with 400 µl 1 M Na_2_CO_3_ and the reaction time was recorded. The cells were centrifuged to sediment debris and OD was measured at A_420_. β-galactosidase activity was calculated using the formula 1000 *(A_420_/(t * v * A_600_ )) where t is the reaction time in minutes and v is the volume of culture assayed in milliliters.

### Protein Expression, Purification and Antibody Production

The anti-PICC/PICL antibody (OSU272) was generated against a partial recombinant protein (PICL amino acids 1 to 100). The N-terminal 6x His-tagged protein was purified from *Escherichia coli* BL21-AI using Ni-NTA resin according to the QIAexpressionist manual (Qiagen, Valencia, CA, USA) and preparative SDS-PAGE. The rabbit antiserum was generated by Cocalico Biologicals, Reamstown, PA, USA.

### Immunoblot Analysis

Arabidopsis protein extracts were prepared by grinding tissues in liquid nitrogen by mortar and pestle and resuspending 100 µl of frozen tissue power in 100 µl of extraction buffer (50 mM Tris-HCl pH 7.5, 150 mM NaCl, 0.5% Nonidet P-40, 1 mM EDTA pH 8.0, 3 mM DTT, 1 mM PMSF, and 1x protease inhibitor cocktail (Sigma-Aldrich)). The samples were centrifuged at 20,000 g for 10 minutes at 4°C to sediment the debris. 3x SDS protein loading buffer was added and the samples were boiled for 5 min. The samples were separated by 8% SDS-PAGE and transferred to PVDF membrane (Bio-Rad, Hercules, CA, USA). The membrane was blocked overnight at 4°C with 4% milk (fat-free dry milk powder) in 1x TBST (50 mM Tris-HCl, 150 mM NaCl, 0.05% tween 20, pH 8.0) and then probed with anti-PICC/PICL antibody (1∶2000) in 1x TBST for 1 h at room temperature. After three washes for 10 minutes each with 1x TBST, the membrane was incubated with anti-rabbit peroxidase conjugated secondary antibody (1∶20,000 in TBST, GE Healthcare, Waukesha, WI, USA) for 1 h. The membrane was again washed three times for 10 min each with 1x TBST and the signals were visualized with SuperSignal West Pico Chemiluminescent Substrate (Thermo Scientific, Waltham, MA, USA) according to the manufacturer’s instructions. Signals were detected on an Optimum Brand X-Ray film (Life Science Products, Frederick, Colorado, USA) using a Konica Minolta Medical Film Processor SRX-101A (Konica Minolta, USA).

### Subcellular Fractionation

Membrane proteins were fractionated essentially as described [39]. Briefly, one hundred milligram of tissue was homogenized in 1 ml of extraction buffer (10 mM Tris-HCl pH 7.0, 0.33 M sucrose, 1 mM EDTA, and 1×plant protease inhibitor cocktail (Sigma-Aldrich)). The homogenate was centrifuged at 20,000 g for 20 min at 4°C to sediment the debris. The supernatant constituted the total (T) fraction. 10 µl of 1 M CaCl_2_ was added to 500 µl of the total fraction and incubated for 1 h on ice. The microsomal fraction (M) was obtained by centrifugation of the total fraction at 25,000 g for 90 min at 4°C. The supernatant was removed and constituted the soluble (S) fraction. The sediment was dissolved in 30 µl extraction buffer and constituted the microsomal fraction (M). 3x SDS protein loading buffer was added, samples were heated at 65°C for 15 min and subjected to 8% SDS-PAGE and subsequent immunoblot analysis with anti-PICC/PICL antibody as described above.

### Post-germination Growth Assays

WT and mutant seeds were sterilized in 40% hypochlorate solution in ethanol, rinsed 6 times in 95% ethanol and dried on a sterile filter paper in a sterile laminar flow hood. The seeds were plated on MS media (1x MS, 0.5% sucrose, 0.5 gl^−1^ MES, 1x Gamborg’s vitamins (Sigma) and 0.8% agar) containing 0, 1.2, 1.4 or 1.6 µM ABA, transferring them individually with a sterile toothpick to ensure even spacing. The plated seeds were vernalized at 4°C for 48 h in the dark. The plates were then placed horizontally in a growth chamber set at 22°C under long day (16 h light/8 h dark) conditions. The percentage of green and expanded cotyledons was calculated by visual inspection at 9 days after vernalization.

### Bacterial Growth Assays

Assays to determine the growth of the T3SS-deficient mutant of *Pst*DC3000 (*hrcC*) and *Pst*DC3000 in WT and mutant Arabidopsis plants were carried out essentially as described [40]. Briefly, suspensions of 1×10^5^ CFU ml^−1^ *Pst*DC3000 or *hrcC* were syringe infiltrated into the lower epidermis of rosette leaves of 5-week-old plants. After infiltration, the leaves were allowed to dry and were subsequently covered with a clear plastic dome to maintain 100% humidity throughout the rest of the experiment under standard growth conditions. After 4 days, nine leaf discs for each infiltration were collected, divided equally into three tubes containing 200 µl 10 mM MgCl_2_ each, ground with pestles and serially diluted to measure bacterial numbers. For flg22-protection assays, 1 µM flg22 peptide was infiltrated into leaves 24 h prior to infiltration with 1×10^5^ CFU ml^−1^ of *Pst*DC3000 and growth was subsequently measured as described.

### RNA Isolation and Quantitative Real-time PCR

For gene expression studies in seedlings, RNA was isolated from 10-day-old seedlings grown in liquid MS media and treated with 1 µM flg22 (10 µl of 1 mM flg22 added to 10 ml of liquid MS media) or water (10 µl of water added to 10 ml of liquid MS media) for 0 h, 1 h and 2 h respectively. For expression studies in Arabidopsis plants, leaves of 5-week-old Arabidopsis plants grown in short day conditions were syringe infiltrated with 1 µM flg22 or 1×10^8^ CFUml^−1^ *hrcC* or water (mock for flg22) or 10 mM MgCl_2_ (mock for *hrcC*). Total RNA was isolated using the RNeasy plant mini kit (Qiagen) and treated with DNase I (Invitrogen).

RNA was quantified using a Nanodrop (Thermo Scientific). cDNA was synthesized from 1 µg of RNA with the Thermoscript RT cDNA synthesis kit (Invitrogen) using an oligo-dT primer. Quantitative real-time PCR was performed on a CFX96TM Real-Time PCR detection system (Bio-Rad) at the PMGF using the iQ™ SYBR Green Supermix (Bio-Rad). qPCR data was analyzed using the CFX96 software (Bio-Rad) and the graphs were generated using the GraphPad Prism software. P-values were calculated based on two-tail non-parametric test (Mann-Whitney test) using the GraphPad Prism software. Actin was used as a control and the primers used for real-time PCR analysis are listed in Table S3.

### ROS Accumulation

ROS accumulation measurements were performed as described [39]. 10–12 leaf discs from 4-week-old plants were excised and floated on distilled water overnight. Three leaf discs each were then transferred into a tube containing 100 µl luminol solution Immun-Star HRP substrate (Bio-Rad), 1 µl of horseradish peroxidase-streptavidin (Jackson Immunoresearch, West Grove, PA, USA) and 1 µl of 1 mM flg22 or water (“mock”). Luminescence was measured using a Glomax 20/20 luminometer (Promega, Fitchburg, Wisconsin) every 10 s until 100 readings were recorded. Three technical repeats were performed for each genotype and treatment (flg22 or mock). The experiment was reproduced in three biological replicates. The values were calculated with luminescence intensity of WT set to 100.

### Computational Analysis

Coiled-coil predictions were carried out using Multicoil [41]. Transmembrane domain predictions were carried out using the TMHMM server v.2.0 [42]. Sequence alignments were generated using the MUSCLE sequence alignment server [43] and the alignment figure was generated using TEXshade [44]. The phylogenetic tree was generated using Phylogeny.fr [45].

## Results

### Identifying Putative Membrane-associated Long Coiled-coil Proteins in Arabidopsis

**Figure 1:**
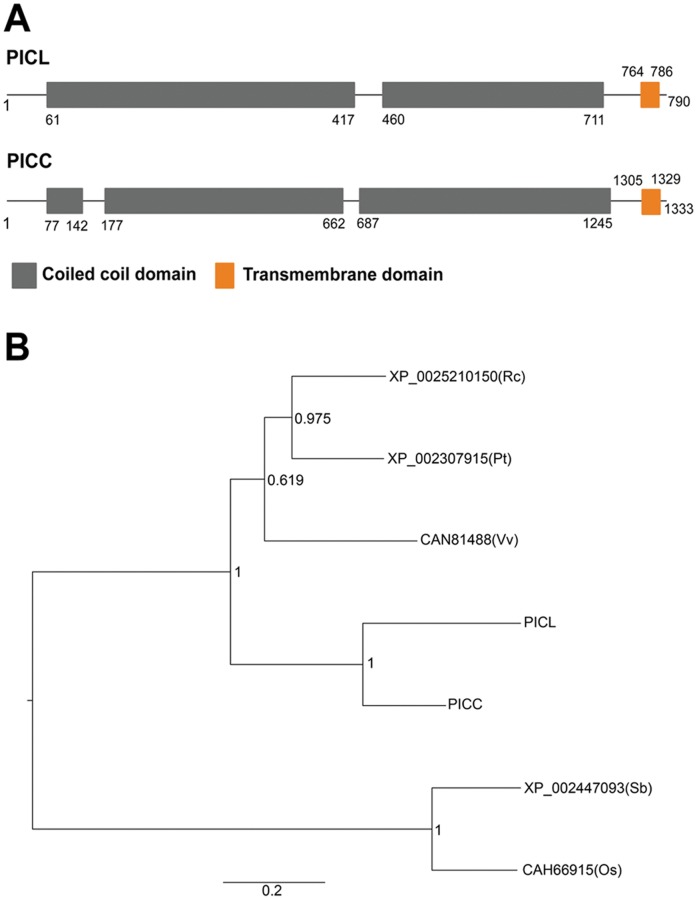
Protein structure of PICC and PICL, and phylogenetic tree of PICC, PICL and their orthologs in vascular plants. (**A**) Putative protein structure of PICC and PICL showing coiled-coil and transmembrane domains. (**B**) Graphical representation of the maximum-likehood phylogenetic tree of PICC, PICL and their orthologs. This phylogenetic tree is based on the multiple sequence alignment shown in Figure S1B. Branch support values are indicated at the nodes as calculated by the PhyML program using default parameters. Os, *Oryza sativa*; Pt, *Populus trichocarpa;* Rc, *Ricinus communis;* Sb, *Sorghum bicolor;* Vv, *Vitis vinifera.*

PICC and PICL were identified in a genome-wide screen for Arabidopsis long coiled-coil proteins with one or more putative transmembrane domains (TMDs). The ARABI-COIL database was used to identify and sort long coiled-coil proteins in the predicted Arabidopsis proteome [15]. The filter parameters were set to identify genes encoding proteins that are at least 500 amino acid long with at least 25% coiled-coil coverage and containing at least one predicted transmembrane domain [15]. Among the fourteen predicted proteins that were identified using the above criteria, PICC, encoded by *At2g32240*, has the highest coiled-coil coverage (79.5%). A close homolog PICL, encoded by *At1g05320*, was also identified based on the above-described criteria. PICC and PICL share 50% identity and 63% similarity at the amino acid level (Fig. S1A). Querying the protein basic local alignment search tool (BLAST) non-redundant (nr) database, orthologs of PICC and PICL were found exclusively in vascular plants and no orthologs were found in non-vascular plants and non-plant organisms (Fig. S1B). Based on the phylogenetic relationships shown in Fig. 1B, it is not possible to determine whether the orthologs in other plant species are more closely related to PICC or PICL.

### PICC and PICL are Located at the ER

**Figure 2:**
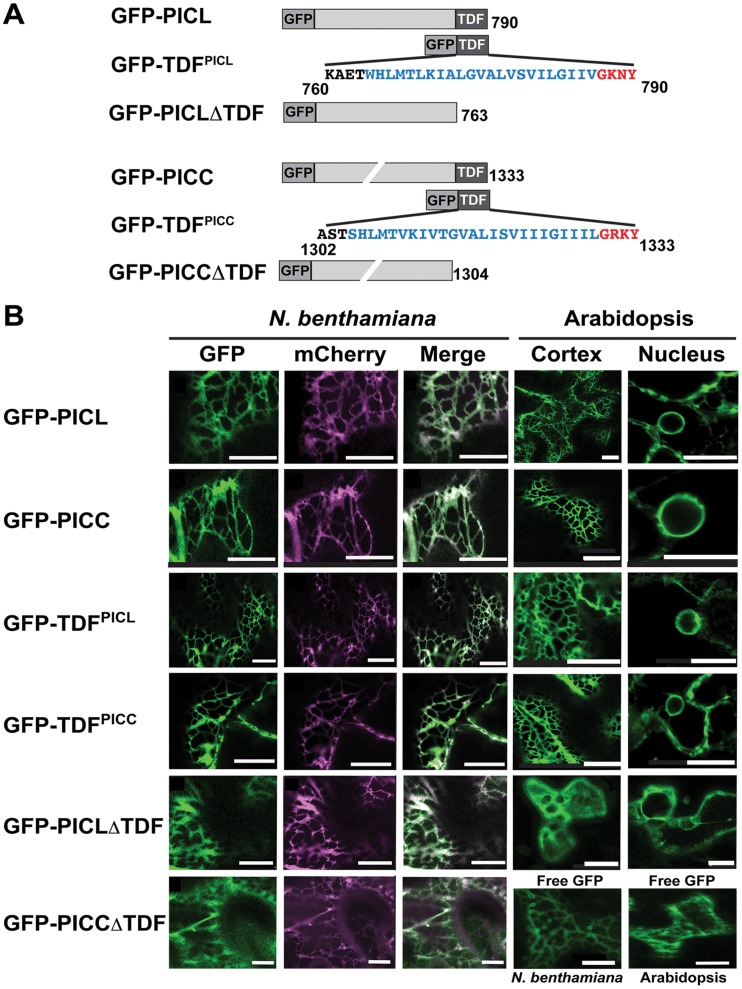
PICL and PICC are associated with the ER via their C-terminal transmembrane domain. (**A**) N-terminally tagged GFP-fusion proteins used in this study. Amino acid sequence of the transmembrane domain and the C-terminal tail are shown in blue and red letters, respectively. Numbers indicate amino acid positions. Drawings are not to scale. (**B**) Confocal images showing localization of the fusion proteins indicated on the left in *N. benthamiana* and Arabidopsis leaf epidermal cells. Cytoplasmic localization of unfused GFP (“Free GFP”) in *N. benthamiana* and Arabidopsis are shown as controls (bottom right). Scale  = 10 µm.

The predicted TMD in PICL and PICC is located 4 amino acids from the C-terminus (Fig. 1A). To investigate the subcellular location of PICC and PICL, GFP-PICC and GFP-PICL fusion proteins were transiently expressed under the control of the Cauliflower mosaic virus *35S* promoter in *Nicotiana benthamiana* leaf epidermal cells and GFP fluorescence was observed by confocal laser scanning microscopy. The proteins were coexpressed with the ER marker HDEL-mCherry. GFP-PICC and GFP-PICL labeled a sharp reticulate network pattern and colocalized with the ER marker (Fig. 2B). When GFP-PICC and GFP-PICL were coexpressed with the tubulin and actin markers RFP-MAP4 (microtubule associated protein 4) and RFP-fABD2 (second actin-binding domain of Arabidopsis fimbrin), respectively, no colocalization was observed (data not shown). To test if a C-terminal fragment of 31 amino acids (transmembrane domain fragment, TDF), which contains the transmembrane domain and the 4 amino-acid tail is sufficient for ER localization, the GFP-tagged partial proteins GFP-TDF^PICC^ and GFP-TDF^PICL^ (Fig. 2A) were generated. In addition, GFP-fused partial proteins without the TDF, GFP-PICCΔTDF and GFP-PICLΔTDF (Fig. 2A) were generated. The four proteins were transiently coexpressed with HDEL-mCherry in *N. benthamiana*. GFP-TDF^PICC^ and GFP-TDF^PICL^ colocalized with the ER-marker. In contrast, GFP-PICCΔTDF and GFP-PICLΔTDF were not located at the ER but were found diffusely distributed in the cytoplasm, closely resembling the localization pattern observed with free GFP (Fig. 2B).

To confirm the localization patterns in Arabidopsis, individual transgenic Arabidopsis lines expressing GFP-PICC, GFP-PICL, GFP-TDF^PICC^, GFP-TDF^PICL^ GFP-PICCΔTDF and GFP-PICLΔTDF under the control of the Cauliflower mosaic virus *35S* promoter were created and at least eight independent T1 transgenic plants for each transgene were imaged. Confirming the localization results obtained in the transient expression experiment, GFP-PICL and GFP-PICC showed the typical reticulate ER localization signals. The TDF domains of both proteins were sufficient to target GFP to the ER. Deleting the TDF of PICL abolished the sharp, reticulate localization pattern and led to a pattern very similar to that of free GFP (Fig. 2B). Transgenic lines expressing GFP-PICCΔTDF could not be recovered, possibly either because PICCΔTDF is rapidly degraded or because its expression is deleterious to plants. Taken together, the localization data from *N. benthamiana* and Arabidopsis indicate that PICL and PICC are ER-associated proteins and that the TDF domain is necessary and sufficient for ER localization.

### The N-terminal Long Coiled-coil Domains of PICC and PICL are Cytosolic

**Figure 3:**
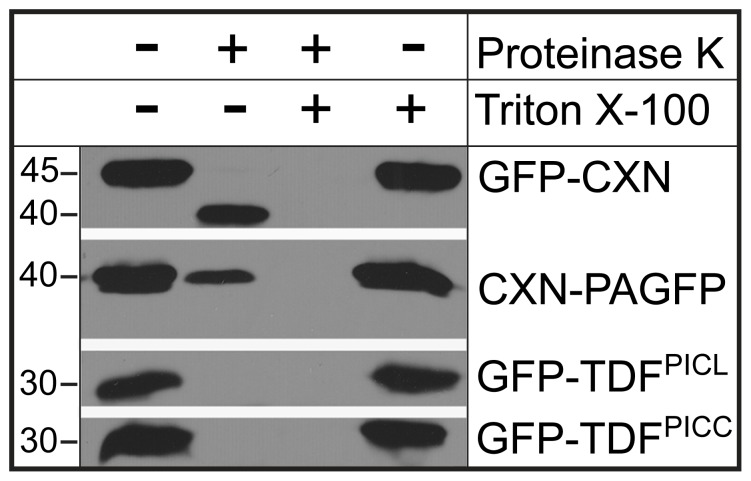
PICL and PICC N-termini face the cytoplasm. Immunoblot analysis using GFP antibody. Microsomal preparations were treated with and without Proteinase K. GFP-CXN and CXN-PAGFP were used as controls. In the microsome fraction containing GFP-CXN, GFP is protected from proteinase K treatment, whereas GFP of CXN-PAGFP is susceptible to proteinase K digestion. GFP of GFP-TDF^PICL^ and GFP-TDF^PICC^ are hydrolyzed indicating exposure to Proteinase K. At the given concentration of proteinase K (sufficient to completely hydrolyze GFP in GFP-TDF^PICL^ and GFP-TDF^PICC^), a small amount of PAGFP remains undigested (second column of CXN-PAGFP). Microsomal membranes were solubilized by the detergent Triton X-100. Numbers on the left indicate approximate molecular mass in kilodaltons.

The position of the single transmembrane domain close to the C-terminus, the absence of any N-terminal signal sequence and the targeting of PICC and PICL to the ER by the TDF indicate that these proteins are tail-anchored (TA) proteins [46], [47]. TA proteins are post-translationally inserted into their target membranes by a single transmembrane domain within the C-terminal 50 residues [47], [48]. TA proteins are characterized by N-terminal functional domains facing the cytoplasm and a short C-terminal tail protruding into the organellar lumen/matrix [46], [47]. In order to determine the topology of PICC and PICL at the ER membrane, a protease protection assay was performed with isolated microsomes. Microsomes were isolated from *N. benthamiana* leaves transiently expressing the fusion proteins GFP-TDF^PICC^ and GFP-TDF^PICL^. Since the localization of GFP-TDF^PICC^ and GFP-TDF^PICL^ is similar to the localization of GFP-PICC and GFP-PICL in both *N. benthamiana* and Arabidopsis (Fig. 2B), the results from this experiment were used to infer the topology of PICC and PICL. ER-resident GFP-fusion proteins with known topology, GFP-Calnexin (GFP-CXN, with GFP facing the ER lumen) and CXN-photoactivatable GFP (CXN-PAGFP, with PAGFP facing the cytoplasm) were used as controls [49], [50]. Immunoblot analysis using an anti-GFP antibody indicated that GFP in GFP-TDF^PICC^ and GFP-TDF^PICL^ was hydrolyzed by proteinase K, whereas the GFP facing the lumen in GFP-CXN was protected from proteinase K (indicated by a band shift due to proteinase K action on the C-terminal cytoplasmic exposed region) and PAGFP facing the cytoplasm in CXN-PAGFP was susceptible to proteinase K digestion (Fig. 3). This demonstrates that the N-terminal GFP tethered to the ER by TDF^PICC^ or TDF^PICL^ is facing the cytoplasm, indicating that the N-terminus of PICC and PICL faces the cytoplasm. Together, these data suggest that PICC and PICL are ER localized, tail-anchored proteins with the N-terminal long coiled-coil domains facing the cytoplasm.

### PICC forms Homodimers and does not Interact with PICL in a Membrane Yeast Two-hybrid System

**Figure 4:**
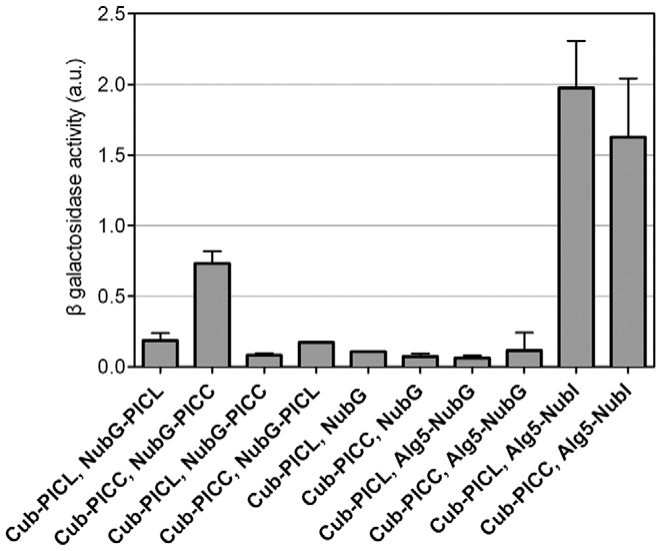
PICC forms homodimers. β-galactosidase activity as a reporter for interaction in a membrane yeast two-hybrid (split-ubiquitin) assay. PICC shows self-interaction as indicated by increased β-galactosidase activity in yeast containing the constructs Cub-PICC and NubG-PICC. β-galactosidase activity in yeast transformed with combinations of Cub-PICL or Cub-PICC with either the empty vector NubG or the unrelated gene Alg5-NubG were used as negative controls. Combinations of Cub-PICL or Cub-PICC with Alg5 fused to wild-type Nub (Alg5-NubI) were used as positive controls. a.u., arbitrary units. Mean values and standard deviation from 3 samples are shown.

Coiled-coil proteins are known to form homo- and hetero-oligomers through specific interactions mediated by their coiled-coil domains [51], [52]. Since PICC and PICL have long coiled-coil domains, the proteins were tested for homo- and heterodimerization using a split-ubiquitin membrane yeast two-hybrid system [53]. In the split-ubiquitin system, an artificial transcription factor (TF) consisting of the LexA DNA binding domain and the VP16 transactivator protein linked to C-terminal moiety of ubiquitin (Cub) is fused to one of the transmembrane proteins and N-terminal moiety of ubiquitin (Nub) is fused to the other transmembrane protein [53]. When expressed alone, the TF-Cub is anchored to the membrane by the transmembrane protein and cannot enter the nucleus to activate reporter genes. In the event of interaction with the protein fused to Nub, a reconstituted ubiquitin moiety is recognized by ubiquitin proteases (UBPs) thus releasing the TF to enter the nucleus and activate the expression of reporter genes. To prevent spontaneous association between Cub and Nub, the Isoleucine at position 13 in wild type Nub (NubI) was changed to Glycine (G) [53]. Therefore, Cub and the mutated Nub (NubG) can only reconstitute upon interaction between two proteins. To test for homo- and heterodimerization, Cub and Nub fusion proteins Cub-PICC, Cub-PICL, NubG-PICC and NubG-PICL were generated. Pairwise interactions of PICC (Cub or NubG) with PICL (Cub or NubG) and homodimerization of PICC and PICL were tested in yeast by measuring the β-galactosidase activity of the LacZ reporter (Fig. 4). The interactions of Cub-PICC with NubG and Cub-PICL with NubG and of Cub-PICC and Cub-PICL with the unrelated protein Alg5-NubG were used as negative controls. The results shown in Fig. 4 indicate that PICC can form homodimers or homo-oligomers, while no evidence for either PICL homodimerization or interaction of PICC with PICL was observed in the yeast split-ubiquitin system.

### PICC and PICL are Expressed in Various Tissues Throughout the Development of the Plant

Using the AtGenExpress Visualization Tool, we analyzed publicly available genome-wide expression data for *PICC* and *PICL* during Arabidopsis development [54]. They indicate expression of both genes in various organs during most stages of plant development, with an overall higher level of expression for *PICC* compared to *PICL* (Fig. S2).

To gain a more detailed impression of the spatial and temporal expression pattern of PICC and PICL, Arabidopsis transgenic plants carrying the reporter gene *β-glucuronidase (GUS*) driven by either 1.0 kb upstream of the start codon (ATG) of *PICL* (*pPICL*) or 2 kb upstream of the start codon of *PICC* (*pPICC*) were generated. The length of the putative promoters *pPICL* (1.0 kb) and *pPICC* (2.0 kb) were chosen based on the presence of other genes and their regulatory elements in the vicinity of *PICC* (nearest gene is *At2g32260* at a distance of 1949 bp from 5′UTR) and *PICL* (nearest gene is *At1g05310* at a distance of 1090 bp from 5′UTR). At least five independent T2 transgenic lines each were analyzed for GUS activity in various organs during different stages of development, from the seedling stage through flowering and maturation of seeds.

**Figure 5:**
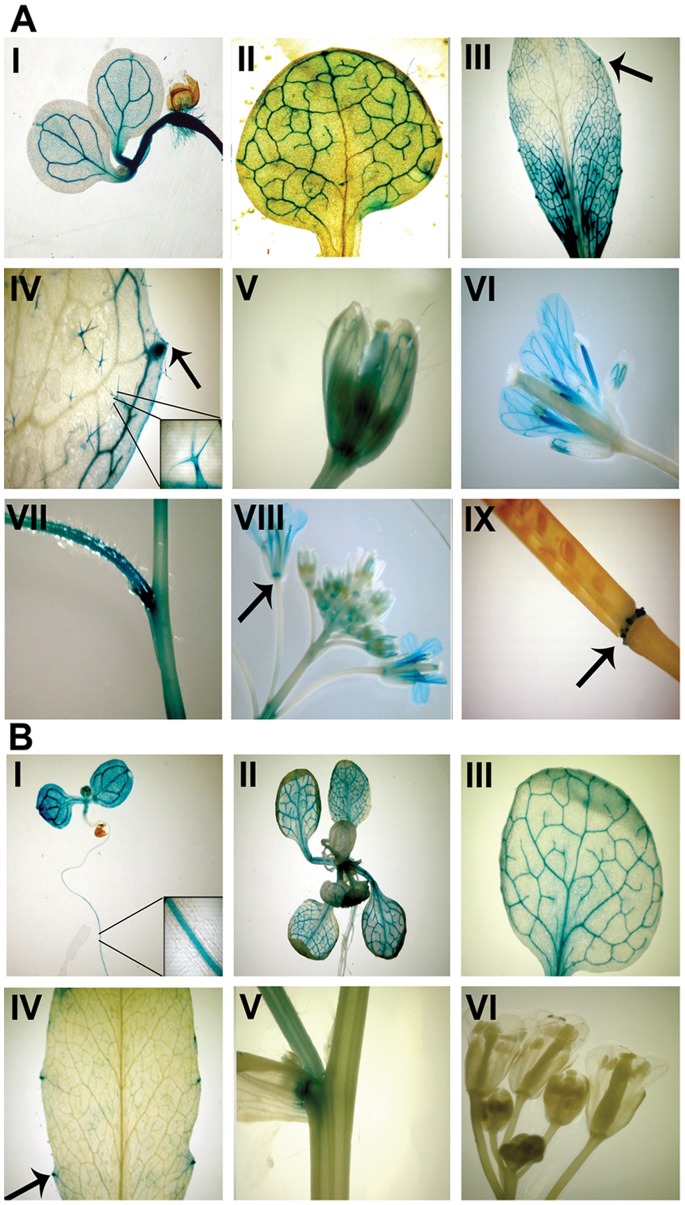
PICC and PICL promoters have partially overlapping patterns of activity. (**A**) β-glucuronidase staining indicating PICC promoter activity in the vasculature of cotyledons, roots, young and mature leaves (I, II and III), in the hydathodes (arrows in III and IV), in the trichomes (IV and inset in IV), in the vasculature of sepals and petals (V and VI), in the filaments of the anther (VI), in the stem and at the nodes (VII) and in the abscission zone of flowers and siliques (arrows in VIII and IX). (**B**) β-glucuronidase staining indicating PICL promoter activity in the vasculature of cotyledons and young leaves (I, II and III), in the vasculature of hypocotyls and roots (I), in the hydathodes (arrow in IV) and at the nodes (V). No activity was visible in the buds, flowers (VI) and siliques (not shown) of the inflorescence.

*pPICL::GUS* expression was detected in the vascular tissue of cotyledons and roots of 7-day-old seedlings, in the vascular tissue of juvenile rosette leaves, in the hydathodes of cotyledons and leaves, and in nodal junctions (Fig. 5B). While *pPICL::GUS* expression was restricted to vegetative organs, *pPICC::GUS* showed a more ubiquitous expression pattern. Similar to *pPICL::GUS*, *pPICC::GUS* expression was detected in the vasculature of cotelydons and roots of 7-day-old seedlings, in the vasculature of juvenile rosette leaves, in the hydathodes of cotyledons and leaves, and in nodal junctions (Fig. 5A). Additionally, expression was observed in leaf trichomes and floral organs. In particular, *pPICC::GUS* expression was seen in the abscission zone at the base of flowers and siliques, in the vasculature of sepals and petals, and in the stamens (Fig. 5A). Taken together, *PICC* and *PICL* have overlapping expression patterns in the vegetative tissues and differential expression patterns in the floral tissues.

### T-DNA Insertion Alleles of *PICC* and *PICL*

A reverse genetics approach was adopted to investigate the function of PICC and PICL. Towards this end, one T-DNA insertion allele for *PICL*, *picl-1* (SALK_56040), and two T-DNA insertion alleles for *PICC*, *picc-1* (SALK_58801) *and picc-2* (SALK_139836) were acquired from the ABRC (Fig. S3A). *picl-1* has a T-DNA insertion in the last (6^th^) exon 58 bp upstream of the region encoding the transmembrane domain. *picc-1* has an insertion within the 2^nd^ exon. *picc-2* also has an insertion within the 2^nd^ exon, 837 bp downstream of the *picc-1* insertion site.

A rabbit polyclonal anti-PICC/PICL antibody, which recognizes both PICC and PICL, was generated using as antigen a 100 aa epitope conserved in both the proteins. The anti-PICC/PICL antibody detects the wild type (WT) PICL protein with an extrapolated mass of ∼90 kDa and the WT PICC protein with an extrapolated mass of ∼160 kDa (Fig. S3B). Immunoblot analysis of protein extracts from the T-DNA insertion lines using the anti-PICC/PICL antibody showed that a truncated PICL (tr.PICL) of ∼80–85 kDa, and a truncated PICC (tr.PICC) of ∼55–60 kDa were produced in *picl-1* and *picc-1* plants, whereas no PICC protein was detected in *picc-2* (Fig. S3B). The bands representing the truncated proteins were weaker than the WT PICL and PICC bands, indicating reduced protein abundance in addition to truncation (Fig. S3B). Moreover, the insertion in *picc-1* results in the loss of ∼2/3^rd^ of the PICC protein and hence, *picc-1* is likely a functionally null allele. However, the insertion in *picl-1* results in only a small C-terminal truncation of PICL. Based on the insertion site in *picl-1*, we predicted that the transmembrane domain is not present in the truncated PICL protein. Subcellular fractionation using total protein extracts of *picl-1* confirmed that tr.PICL is soluble and not associated with membranes (Fig. S3C). The loss of membrane association of tr.PICL in the *picl-1* mutant indicates that *picl-1* is a null allele for functions that require its insertion into the ER membrane. The major truncation and significantly reduced abundance of PICC in *picc-1* and the absence of detectable PICC in *picc-2* suggests that *picc-1* and *picc-2* are likely functionally null alleles.

PICC and PICL are encoded by paralogous genes and have 50% amino acid sequence identity as well as a similar domain structure, localization and topology. Therefore, we predicted that there is a high probability of functional overlap between the two proteins. Thus, a *picc-1;picl-1* double mutant was generated by crossing homozygous *picc-1* and *picl-1* mutant lines. Since *picc-1* was the first allele to be selected, most of our analysis was conducted with the *picc-1* mutant allele alongside *picl-1,* WT and the double mutant *picc-1;picl-1.*

### *picc-1, picc-2, picl-1* and *picc-1;picl-1* Mutant Plants are Hypersensitive to ABA during Post-Germination Growth

**Figure 6:**
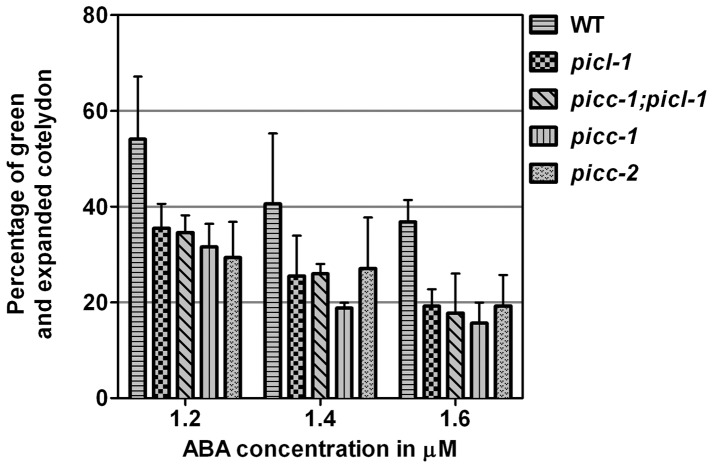
*picl-1*, *picc-1;picl-1, picc-1*, and *picc-2* are hypersensitive to ABA at the post-germination growth stage. WT, *picl-1, picc-1;picl-1, picc-1,* and *picc-2* were grown on MS plates containing different concentrations of ABA. Post-germination growth efficiency was determined as percentage of green and expanded cotyledons at 10 days after stratification. Values represent average of three replicates, where number of seeds  = 54 in each replicate. Error bars represent one standard deviation. On plates containing 0 µM ABA, WT and mutants had 100% post germination growth efficiency. Similar results were obtained in three out of four biological replicates.

The appearance of the single mutants *picc-1*, *picc-2, picl-1* and the double mutant *picc-1;picl-1* was indistinguishable from WT throughout the development of Arabidopsis. Plants in their natural environment are exposed to a variety of abiotic and biotic stress conditions such as drought, high salinity, temperature variations etc. Therefore, we investigated the response of *picc-1, picl-1 and picc-1;picl-1* plants under osmotic and salt stress conditions. Germination and post-germination seedling growth were investigated on medium containing 50 mM, 100 mM or 150 mM NaCl (salt-stress), or 100 mM, 200 mM or 300 mM mannitol (osmotic stress). *picc-1*, *picl-1* and *picc-1;picl-1* germination and seedling growth was indistinguishable from WT (data not shown). To investigate hormonal stress response, WT and mutant *picc-1, picc-2, picl-1* and *picc-1;picl-1* seeds were analyzed for germination and post-germination seedling growth on medium containing 1.2, 1.4, and 1.6 µM abscisic acid (ABA). Although the rate of germination of all the mutants was similar to WT, all the mutant plants showed hypersensitivity to ABA during the post-germination growth (Fig. 6). *picc-1, picc-2, picl-1* and *picc-1;picl-1* showed a lower percentage of green and expanded cotyledons compared to the WT (Fig. 6) indicating a modulation in the post-germination growth response to ABA.

### ER Morphology is not Altered in *picc-1;picl-1* Mutant Plants

Since GFP-PICC and GFP-PICL are localized at the ER in Arabidopsis, we investigated the ER morphology in *picc-1;picl-1* double mutant plants. Towards this end, we transformed *picc-1;picl-1* and WT plants with the ER marker HDEL-mCherry, driven by the Cauliflower mosaic virus *35S* promoter, and imaged mCherry fluorescence using confocal laser scanning microscopy. The morphology of the cortical ER was highly similar in WT and *picc-1;picl-1*. Thus, there is currently no evidence for an involvement of PICC and PICL in ER organization (Fig. S4).

### *PICC* Expression is Activated by the Bacterial Elicitor Flagellin 22 (flg22)

**Figure 7:**
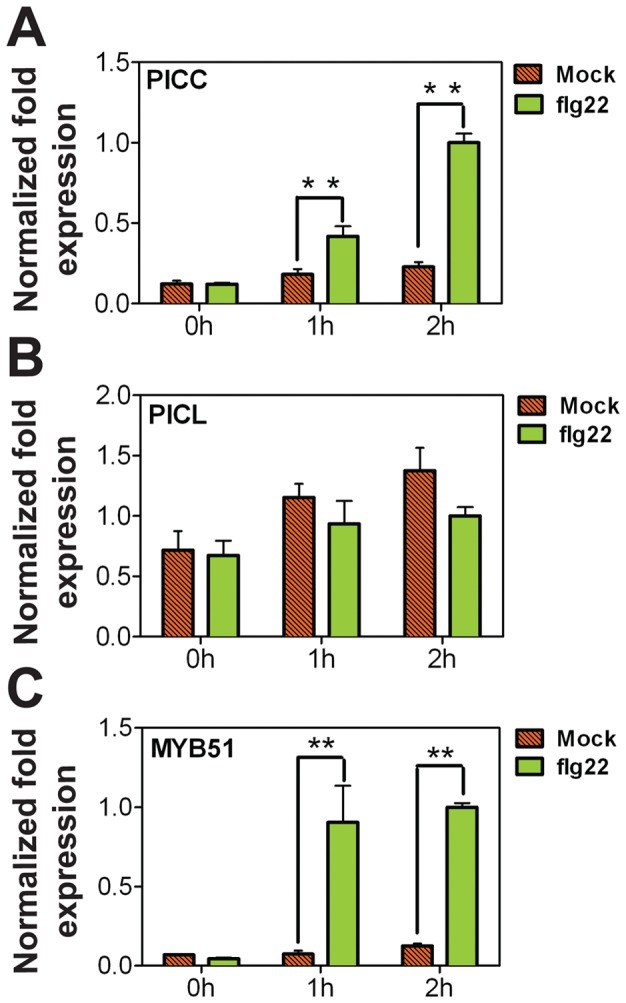
PICC expression is induced by flg22. 10-day-old liquid-grown seedlings were treated with water or flg22. *PICC* (**A**) *and PICL* (**B**) steady-state mRNA levels were quantified by real-time RT-PCR at times indicated. (**C**) *MYB51,* a known flg22-induced gene, was used as a positive control. Transcript levels were normalized to *ACTIN* measured in the same samples. Values are given in arbitrary units with expression in 2 h flg22 treated samples set to 1. Each value is represented as the average of two biological replicates. Error bars represent one standard deviation. Double asterisks (**) indicate statistically significant difference in values compared to mock treated samples at the corresponding time point (P<0.01).

Based on expression analysis of public microarray data (affymetrix ATH1) using the Genevestigator database and analysis tools [55], *PICC* expression appeared to be upregulated after treatment with flg22. Recognition of PAMPs on the bacteria by plant PRRs induces global transcriptional changes in the plant. To confirm the induction of *PICC* expression by flg22, quantitative RT-PCR analysis was performed on RNA extracted from seedlings and the expression of *PICC* and *PICL* was analyzed after 0, 1 and 2 h continuous treatment with flg22. Consistent with the public microarray data, *PICC* was induced after 1 h and induction was further increased after 2 h of flg22 treatment, whereas *PICL* expression was not changed (Fig. 7A and B). The transcription factor *MYB51* was included as a positive control gene known to be strongly induced by flg22.

**Figure 8:**
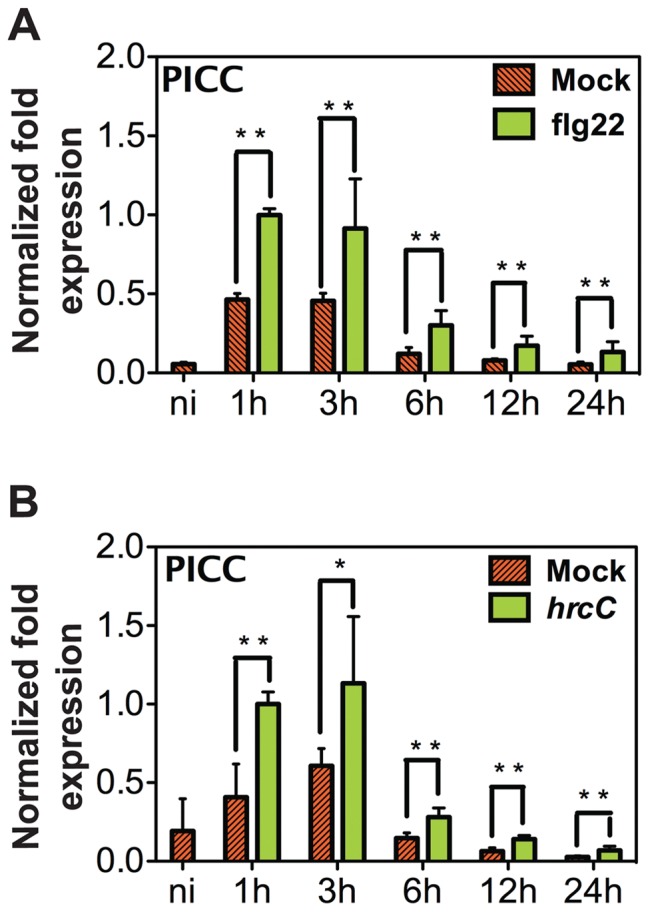
Time course of *PICC* induction. 4-week-old WT Col-0 plants were infiltrated with 1 µM flg22 (**A**), or 2×10^8^ CFU ml^−1^ type III secretion deficient *hrcC* (**B**). *PICC* steady state mRNA levels were quantified by real-time PCR at times indicated. Transcript levels were normalized to *ACTIN* levels from the same sample. Values are given in arbitrary units with the value in 1 h flg22 treated sample set to 1. Each value is represented as the average of three biological replicates for treatment with flg22 (A) and *hrcC* (B). Error bars represent one standard deviation. Double (**P<0.01) and single (* P<0.05) asterisks indicate statistically significant difference in values compared to mock treated samples at the corresponding time point.

To investigate whether *PICC* expression is also induced by bacterial infection, rosette leaves of 5-week-old plants grown in short day conditions were syringe-infiltrated with either 1 µM flg22 or the avirulent *P. syringae* strain *Pst*DC3000 *hrcC (hrcC)* or water (mock for flg22) or 10 mM MgCl_2_ (mock for *hrcC*) and the expression of *PICC* and *PICL* was analyzed at 1, 3, 6, 12 and 24 hours post infiltration (hpi). *PICC* was induced by mock treatments peaking at 1 hpi, suggesting wounding may induce this gene. However, *PICC* induction at 1 hpi with both *hrcC* and flg22 treatments was significantly higher than with mock treatment (Fig. 8). While *PICC* expression induced by mock treatment gradually decreased reaching basal levels at 24 hpi, *PICC* induction by flg22 and *hrcC* continued to remain significantly higher than the mock induction during all time points tested (Fig. 8). Consistent with the microarray data analysis and expression analysis in seedlings, *PICL* was not induced after flg22 and *hrcC* treatments (data not shown) confirming that *PICC* and *PICL* are differentially regulated during plant defense response.

### *picc-1* Mutant Plants are More Susceptible to *Pst*DC3000 *hrcC*

**Figure 9:**
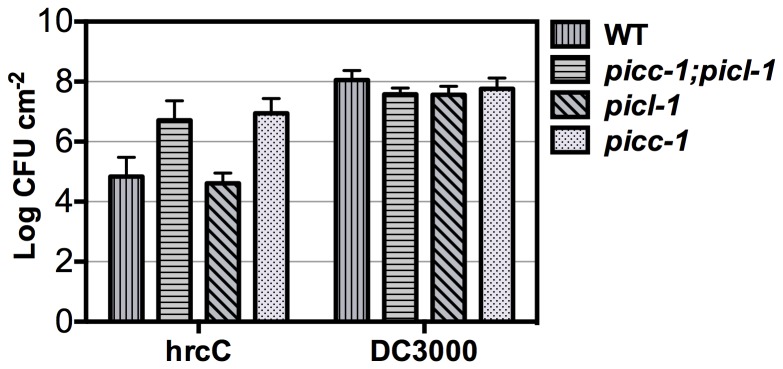
*picc-1* and *picc-1;picl-1* Arabidopsis plants are more susceptible to *hrcC*. Levels of type III secretion deficient *hrcC* and wild-type *Pst*DC3000 four days after infiltration into leaves of the indicated plants. Values represent average of three replicates. Error bars represent one standard deviation. Increased growth of *hrcC* in *picc-1* and/or *picc-1;picl-1* relative to WT was observed in 6 out of 7 biological replicates. CFU, Colony Forming Units.

Based on the PAMP-induced *PICC* expression, we hypothesized that *picc-1* mutant plants may be more susceptible to avirulent *hrcC* bacteria. To test this hypothesis, the growth of the virulent pathogen (*Pst*DC3000) and the nonvirulent pathogen *hrcC* was analyzed in WT, *picc-1*, *picl-1* and *picc-1;picl-1* plants. Rosette leaves were syringe-infiltrated with 10^5^ colony-forming units (CFU) of bacterial suspensions of *hrcC, Pst*DC3000 or 10 mM MgCl_2_ (mock). Growth of bacteria was measured at 4 days post infiltration. *picc-1* and *picc-1;picl-1* plants supported as much as 100–150 fold greater *hrcC* growth compared to WT, while *picl-1* plants behaved like WT (Fig. 9). In addition to *picc-1*, we observed similar enhanced susceptibility to *hrcC* in the *picc-2* mutant (Fig. S5). These results indicate a role for PICC in defense against *hrcC*. Contrary to the result with *hrcC,* no significant difference in the growth of *PstDC3000* was observed between WT and any of the mutant plants (Fig. 9 and Fig. S5). We also conducted flg22-protection assay in which infection with *Pst*DC3000 is preceded by infiltration with the flg22 peptide. WT, *picl-1*, *picc-1* and *picc-1;picl-1* mutant plants were syringe-infiltrated with 1 µM flg22 or water (mock) and 24 h later were infiltrated with 10^5^ CFU of *Pst*DC3000. Bacterial growth was then assessed after 4 days. Growth of *Pst*DC3000 was equally reduced in all genotypes pre-treated with flg22 compared to growth in mock-treated plants indicating that this assay does not reveal compromised flg22-induced defenses in *picc-1*, *picl-1* and *picc-1;picl-1* mutant plants (Fig. S6).

To investigate the effectiveness of PTI signaling in *picc-1* mutant plants and to narrow down the point of action of PICC, we analyzed different PTI responses. PAMP perception triggers a rapid burst of reactive oxygen species (ROS) [2]. ROS generation occurs as an early response to PAMPs and is one hallmark of successful pathogen recognition and activation of defense responses [56]. We examined ROS production following flg22 treatment in *picc-1* and WT plants using a luminol-based assay. *picc-1* plants did not show any significant difference in ROS accumulation compared to WT plants indicating that PICC is not involved in flg22-induced accumulation of ROS (Fig. S7).

To further investigate PAMP responses, we examined PAMP-induced gene expression changes by Q-RT PCR. Rosette leaves of WT and *picc-1* mutant plants were treated with flg22 or *hrcC* or water (mock for flg22) or 10 mM MgCl_2_ (mock for *hrcC*) and mRNA was analyzed at 1, 3, 12 or 24 hpi. *MYB51* is an early PAMP-induced transcription factor essential for cell wall-reinforcing callose deposition at the sites of infection. Suppression of callose deposition is associated with increased growth of *hrcC.* As expected, *MYB51* induction was greater with flg22 and *hrcC* than with mock treatments (Fig. S8A). No significant difference in *MYB51* induction was found between *picc-1* and WT plants, indicating that PAMP-induced *MYB51* expression is not affected by loss of *picc-1* (Fig. S8A). Analysis of the basal level of *MYB51* indicated a slight increase in *picc-1* mutants compared to WT; however, the difference was not statistically significant.

Next, we examined PAMP-induced salicylic acid (SA) accumulation and signaling through gene expression analysis of the SA biosynthesis gene *isochorismate synthase (ICS1)* [57] and the classic read-out for SA signal transduction, *PR1* [58]. PAMP-regulated gene expression is partially dependent on PAMP-triggered SA accumulation which is important for PTI [59]. *ICS1* and *PR1* expression was analyzed at 12 and 24 hpi. *ICS1* expression was greater with flg22 and *hrcC* treatments at 12 and 24 hpi than with mock treatments in WT and *picc-1* (Fig. S8B). However, no significant change was observed in *picc-1* mutants compared to WT (Fig. S8B). Similarly, *PR1* expression levels did not show any difference in *picc-1* plants compared to WT indicating that SA signaling leading to PR1 gene expression is not compromised in *picc-1* plants (Fig. S8C).

Previous studies have established a role for ABA in pathogen response. An increase in ABA levels increases a plant’s susceptibility to pathogens [60], [61], [62]. 9-*cis*-epoxycarotenoid dioxygenase 3 (NCED3) is a key enzyme in stress induced ABA biosynthesis pathway [63]. In light of the increased ABA-sensitivity in the post-germination response of *picc* and *picl* mutants, we tested if *NCED3* expression levels are affected in the *picc-1* mutant. The levels of *NCED3* transcript were investigated at 1, 3, 12 and 24 hpi after flg22 and *hrcC* treatments. *NCED3* expression levels showed no significant difference between wild type and *picc-1* mutants, thus excluding a scenario of increased ABA levels in *picc-1* resulting from an increase in the expression of NCED3 (Fig. S8D).

Collectively, these data indicate that the branches of PTI leading to ROS production, and accumulation of *MYB51*, *ICS1*, *PR1*, and *NCED3* transcripts are not compromised in *picc-1* mutant plants.

## Discussion

### PICC and PICL are Plant-specific, ER-associated Long Coiled-coil Proteins

Long coiled-coil proteins play an important role in various cellular processes and function as scaffolds and platforms for tethering cellular functions. In this work, we have characterized a family of two plant-specific long coiled-coil proteins in Arabidopsis, PICC and PICL. Interestingly, only 14 predicted membrane proteins in Arabidopsis contain long coiled-coil domains [15] and among them, PICC has the highest percentage (79.5%) of amino acids that can form coiled-coil domains, followed by PICL (63.7%). Based on sequence similarity, we could identify putative orthologs only in plants, indicating that PICC and PICL may be involved in plant-specific processes (Fig. 1B).

PICC and PICL are localized at the ER. Confocal microscopic analysis investigating the localization of truncated proteins showed that the transmembrane domain fragment (comprised of transmembrane domain and tail) is necessary and sufficient for localizing PICC and PICL to the ER. This indicates that the targeting information resides in the C-terminal 31 amino acids (Fig. 2B). The TDF is highly conserved across all plant orthologs, indicating that targeting information and hence the targeting mechanism is conserved. Thus, ER localization is likely important for PICC/PICL protein function. Dissecting the TDF by mutational analysis will further reveal whether the ER targeting information is in the tail or in the transmembrane domain region or whether the entire TDF is essential for ER localization.

### PICC and PICL are Tail-anchored (TA) Proteins

The domain organization of PICC and PICL indicates that the proteins are likely targeted to the ER by a tail-anchoring mechanism. TA proteins are a unique class of integral membrane proteins in eukaryotes that are involved in diverse cellular processes [47], [64]. They are post-translationally targeted to their respective organelles by a single transmembrane domain located close to the C-terminus and feature functional N-terminal domains that face the cytoplasm [47], [64]. Consistent with the requirements for TA proteins, domain analysis by confocal microscopy showed that the transmembrane domain and the tail of PICC and PICL are necessary and sufficient to target the proteins to the ER (Fig. 2B). A protease-protection assay showed that the N-termini of PICC and PICL are facing the cytoplasm (Fig. 3). These studies confirm that PICC and PICL are indeed TA proteins, targeted to the ER by information contained in the C-terminal 31 amino acids. Supporting our analysis, PICC and PICL were identified along with ∼520 other proteins in two bioinformatic screens for TA proteins in Arabidopsis [46], [48].

While it is possible to predict TA proteins using bioinformatics based on the simple definition above, accurately predicting their localization is a challenging task. Bioinformatic tools are able to predict correct TA protein localization to the ER in only 62% of cases [48]. Hence, the transmembrane domain fragments of PICC and PICL can now serve as valuable tools to dissect the importance of individual residues for ER targeting, with the goal to establish more stringent and relevant criteria for predicting TA protein localization in plants. Multiple pathways have been described for targeting TA proteins to the ER membrane in animals and yeast [48]. However, very little is known about the biogenesis of TA proteins in plants, largely due to a much smaller number of TA proteins that have been experimentally characterized [48]. PICC and PICL can thus serve as new candidate client proteins for approaches to identify and characterize putative plant ER tail-anchoring machinery.

### Differential Regulation during Development

Promoter::GUS analysis of upstream regulatory regions of PICC and PICL suggest that these proteins are differentially regulated during development. However, they show partially overlapping expression patterns in the vasculature of cotelydons and leaves, in roots of seedlings and in hydathodes (Fig. 5A and B). *PICC* promoter::GUS activity, in addition, is observed in leaf trichomes, in the vasculature of sepals and petals, in stamen filaments and in the abscission zone at the base of the siliques and flowers (Fig. 5B). The differential expression of *PICC* and *PICL* promoters during development indicates that these paralogous proteins may function in different cellular processes. Hydathodes are highly specialized pores positioned at the leaf margins [65]. They mediate secretion of sap containing ions, metabolites and proteins through a process called guttation [65], [66]. However, they lack physical barriers and are convenient routes for pathogen entry. *Xanthomonas campestris,* the bacteria responsible for black rot in cabbage, enters the plant apoplast mainly through the hydathodes [67], [68]. Immune responses such as lignification of hydathodes has been observed after *X.campestris* infection [68]. PR proteins such as chitinases are expressed in hydathodes also, presumably as a preventive mechanism for restricting pathogen entry in the absence of physical barriers [69], [70], [71]. *PICC* is also expressed in the floral abscission zone. The Arabidopsis transcription factors, AtWRKY6 and AtWRKY33, associated with abscission and defense response, are expressed in the abscission zone [72], [73]. Microarray analysis of tomato and citrus abscission zone transcriptomes showed preferential expression of defense related genes [74], [75]. These studies prompt us to speculate that the constitutive expression of *PICC* in hydathodes and the abscission zone may function to preempt pathogen entry in these disease-vulnerable zones.

### Differential Expression in Response to Biotic Stimuli

Recognition of pathogens by plants activates complex signal transduction mechanisms leading to global transcriptional reprogramming. Among the genes induced by PAMP recognition are those that encode proteins involved in signal perception and transduction, transcriptional regulation, synthesis and delivery of antimicrobial compounds [76], [77], [78], [79]. The increase in *PICC* gene expression at 1 h (earliest time point tested) post treatment with either flg22 or *hrcC* and the persistence of induction for at least 24 h indicate that *PICC* is an early PAMP-induced gene (Fig. 8). In contrast, the expression level of *PICL* was not affected by flg22 or *hrcC*. The induction of *PICC* gene is consistent with a role for PICC in PTI, which is evidenced by an increased growth of *hrcC* bacteria in *picc-1* mutant plants compared to WT plants (Fig. 9). While phenotypic evidence clearly points towards a role for PICC in PTI, *picl-1* mutant plants behaved like WT plants after *hrcC* infection (Fig. 9). *picl-1* mutant plants produce truncated PICL protein at almost WT levels, which is no longer associated with the membrane (Fig. S3C). Therefore, the absence of increased susceptibility of *picl-1* mutant plants to *hrcC* could be due to the presence of a partly functional cytoplasmic truncated PICL protein (Fig. S3C). In this scenario, ER localization might not be essential for the function of PICC and PICL in plant defense response.

Based on the PAMP-induced gene expression and the *hrcC* growth phenotype, we suggest that PICC might play a role in PTI in Arabidopsis. Unlike *PICC*, *PICL* is not induced by PAMPs, indicating that, at least at the level of regulation, these duplicated Arabidopsis genes differ with respect to their role.

While two *PICC* T-DNA insertion alleles were analyzed (*picc-1,* Fig. 6; Fig. 9, and *picc-2,* Fig. 6; Fig. S5), only one *PICL* T-DNA insertion allele was tested (*picl-1*, Fig 6; Fig 9). Thus, it cannot be excluded at the present time that the phenotypes observed are influenced by second-site mutations in the respective mutant backgrounds. Future work, involving complementation of *picc-1* and *picc-2* with PICC or GFP-PICC, and complementation of *picl-1* with PICL or GFP-PICL, driven by their native promoters, will unequivocally resolve this question.

### Relationship with ABA

While the role of PICL in defense response is not established, the ABA hypersensitivity of *picc-1, picc-2* and *picl-1* mutants during post-germination growth suggests that both PICC and PICL might play a role during ABA-induced stress (Fig. 6). Increased sensitivity to ABA in the mutant plants could be due to either increased levels of endogenous ABA or due to enhanced ABA signaling. The role of ABA in response to abiotic stresses such as drought, salinity and cold is well established [80], [81]. ABA also plays an important role in modulating plant defense response. ABA functions antagonistically with SA and negatively regulates defense response to pathogens [62], [82]. Increased ABA levels correlate with increased virulence of pathogens [60]. NCED3 is a key enzyme in stress-induced ABA biosynthesis [63]. Analysis of *NCED3* expression levels in WT and *picc-1* mutant plants after flg22 treatment and *hrcC* infection showed no change (Fig. S8D), indicating that the increased growth of *hrcC* in *picc-1* is not due to increased ABA biosynthesis from increased expression of the *NCED3* gene. This does not rule out the possibility of increased endogenous ABA levels in *picc-1* plants, which could possibly result in compromised PTI, since an increase in endogenous ABA levels has been associated with increase in growth of pathogens [60]. However, if the ABA hypersensitivity of *picc-1* is associated with the compromised PTI, the question remains why *picl-1*, which is also hypersensitive to ABA, does not show increased susceptibility to *hrcC*. Further work that involves analysis of defense response of *picc-1*, *picc-2* and *picl-1* mutants in ABA-deficient or ABA-hypersensitive backgrounds and analysis of ABA response during pathogen infection in *picc-1*, *picc-2* and *picl-1* mutants will yield better insights into the function of PICC and PICL in hormonal response and its correlation with the innate immune response.

### Dissecting PTI Signaling Pathways

The rapid production of ROS and induction of ethylene biosynthesis occur as early responses to successful pathogen recognition and activation of defense responses [2]. Ethylene signaling is important for maintaining FLS2 levels on the plasma membrane and reduced FLS2 levels result in dampened PTI signaling which, in turn, results in reduced ROS production [83]. Additionally, ethylene signaling is important for PAMP-induced expression of the *MYB51* transcription factor, which regulates callose deposition [84]. *picc-1* plants are not compromised in ROS production (Fig. S7) and do not show any change in *MYB51* induction compared to WT (Fig. S8A). Taken together, these results indicate that ethylene signaling is not compromised in *picc-1* plants and that PICC functions in a pathway either parallel or downstream to that of ROS production and induction of *MYB51* expression. Similarly, based on WT-like behavior of *picc-1* with respect to the expression levels of the SA biosynthesis gene, *ICS1*, and the marker for SA signaling, *PR1,* we can place *PICC* in a parallel or a downstream pathway to SA signaling.

While the pathway of PICC action is still unknown, it is possible to speculate on a molecular role, based on its coiled-coil nature and preliminary expression analysis. According to expression analysis of public microarray (Affymetrix ATH1) data using the Genevestigator database and analysis tools [55], in addition to PAMP induction, *PICC* expression appears to be upregulated upon infection with the powdery mildew fungus *Bgh* (data not shown). Dynamic reorganization of subcellular components such as actin, microtubule, ER, Golgi apparatus [85] and peroxisomes [86] at the sites of infection have been shown to be important for resistance against both fungal and oomycete pathogens [87]. Focal concentration of components of vesicle trafficking, PEN1, SNAP33 and VAMP721/722, are observed at the sites of fungal infection [88]. However, the molecular mechanisms that recruit the cellular components to the infection sites are unknown. Studies from animals and yeast show that the vesicle fusion events are primed by the tethering of long coiled-coil proteins, which mediate the initial attachment of the carrier vesicles to the target membrane [89], [90], [91]. Similarly, it is possible that molecular tethers formed typically by long coiled-coil proteins function in the cellular reorganization during fungal infection.

Taken together, this study reports a novel relationship between a long coiled-coil protein and plant defense response and suggests a possible role for the PICC-PICL family in coping with hormonal stress during post-germination growth. Phylogenetic analysis indicates that a recent gene duplication event in Arabidopsis has given rise to *PICC* and *PICL* while only one ortholog is present in other plant species. It is thus possible that PICC has recently acquired the defense-related function, which can be addressed by investigating PICC/PICL orthologs in other plant species for their role in PTI as well as in ABA response.

## Supporting Information

Figure S1 **Sequence and phylogeny of PICC and PICL. (A)** Sequence alignment of PICC and PICL. **(B)** Multiple sequence alignment of PICC, PICL and their orthologs in vascular plants. Blue bar below the alignment indicates the predicted transmembrane domain. Os, *Oryza sativa*; Pt, *Populus trichocarpa;* Rc, *Ricinus communis;* Sb, *Sorghum bicolor;* Vv, *Vitis vinifera.*
(DOCX)

Figure S2 **Expression pattern of** ***PICC*** **and** ***PICL*** **in various organs.** *PICC* (At2g32240) and *PICL* (At1g05320) expression pattern based on microarray expression data using the AtGenExpress Visualization Tool [54]. Each point indicates an expression value from an independent experiment [54].
(DOCX)

Figure S3 **T-DNA insertion alleles of PICL and PICC. (A)** Genomic structure of PICL and PICC showing T-DNA insertion sites in *picl-1*, *picc-1* and *picc-2* alleles. Black asterisk (*) indicates the region encoding the antigen (amino acids 1–100) used for anti-PICC/PICL antibody development. This sequence is highly conserved in PICC and PICL. **(B)** Immunoblot analysis using PICL antibody detecting the presence of full-length PICC and PICL and truncated PICL (tr.PICL) and truncated PICC (tr.PICC) in WT and mutant Arabidopsis protein extracts. Molecular mass markers are indicated on the right. Ponceau membrane stained with Ponceau S before immunoblotting, indicating close-to equal loading. The 50 kDa RBCS band is shown. **(C)** Total (T), microsomal (M) and soluble (S) fractions of WT and *picl-1* Arabidopsis leaf protein extracts detected in an immunoblot with the PICL antibody. In WT, PICL is associated with the membrane and is detected in the microsomal fraction. In *picl-1,* the truncated protein lacks the transmembrane domain and is no longer associated with the membrane, which is evident by the absence of truncated PICL in the microsomal fraction. Ponceau-stained membrane is shown for loading control.
(DOCX)

Figure S4 **ER morphology is not visibly altered in** ***picc-1;picl-1*** **mutant plants.** Confocal images of Arabidopsis leaves expressing HDEL-mCherry in WT (A and C) and *picc-1;picl-1* (B and D). Scale  = 10 µm.
(DOCX)

Figure S5 ***picc-1, picc-2*** **and** ***picc-1;picl-1*** **Arabidopsis plants are more susceptible to the avirulent bacterial strain** ***hrcC.*** Levels of *hrcC* and *Pst*DC3000 four days after infiltration into leaves of the indicated plants. Values represent average of three replicates. Error bars represent one standard deviation. CFU, Colony Forming Units.
(DOCX)

Figure S6 **flg22-induced resistance against** ***Pst*** **DC3000 is not compromised in** ***picl-1*** **,** ***picc-1*** **or** ***picc-1;picl-1*** **mutant plants.** Bacterial suspension of *Pst*DC3000 was infiltrated into indicated plants 24 h after pretreatment with water (mock) or 1 µM flg22 (flg22). Values represent average of three replicates. Error bars represent one standard deviation. Similar results were obtained in two biological replicates. CFU, Colony Forming Units.
(DOCX)

Figure S7 **Generation of reactive oxygen species is not compromised in** ***picc-1*** **. (A)** Total ROS generation triggered by 10 µM flg22 in WT and *picc-1* represented as a percentage of WT. Values represent average of three biological replicates. Error bars represent one standard deviation. **(B)** A time trace of the flg22 triggered oxidative outburst in WT and *picc-1*. WT and *picc-1* treated with mock (water) are shown as negative controls. Similar results were obtained in three biological replicates.
(DOCX)

Figure S8 **PAMP-induced expression changes are not altered in** ***picc-1.*** Leaves from 4-week-old WT and *picc-1* plants were infiltrated with 1 µM flg22 or bacterial suspensions (2×10^8^ CFU ml^−1^) of type III secretion deficient *hrcC*. Steady state mRNA levels of **(A)** *MYB51*, **(B)** *ICS1*, **(C)** *PR1* and **(D)** *NCED3* were quantified by real-time PCR at times indicated. Transcript levels were normalized to *ACTIN* levels from the same sample. Values are given in arbitrary units with the value in 24 h WT samples infiltrated with either flg22 or *hrcC* set to 1. Each value is represented as an average of three biological replicates. Error bars indicate one standard deviation.
(DOCX)

Table S1 **Primers used for genotyping.**
(DOCX)

Table S2 **Primers used for cloning.**
(DOCX)

Table S3 **Primers used for real-time PCR.**
(DOCX)
