# Supplementary material for: Functional Investigation of the Plant-Specific Long Coiled-Coil Proteins PAMP-INDUCED COILED-COIL (PICC) and PICC-LIKE (PICL) in Arabidopsis thaliana
Source: PLoS One. 2013 Feb 25;8(2):e57283. doi: 10.1371/journal.pone.0057283 (PMC3581476; doi:10.1371/journal.pone.0057283)
Supplement: Figure S1 — Sequence and phylogeny of PICC and PICL. (A) Sequence alignment of PICC and PICL. (B) Multiple sequence alignment of PICC, PICL and their orthologs in vascular plants. Blue bar below the alignment indicates the predicted transmembrane domain. Os, Oryza sativa; Pt, Populus trichocarpa; Rc, Ricinus communis; Sb, Sorghum bicolor; Vv, Vitis vinifera. (DOCX) [file pone.0057283.s001.docx]

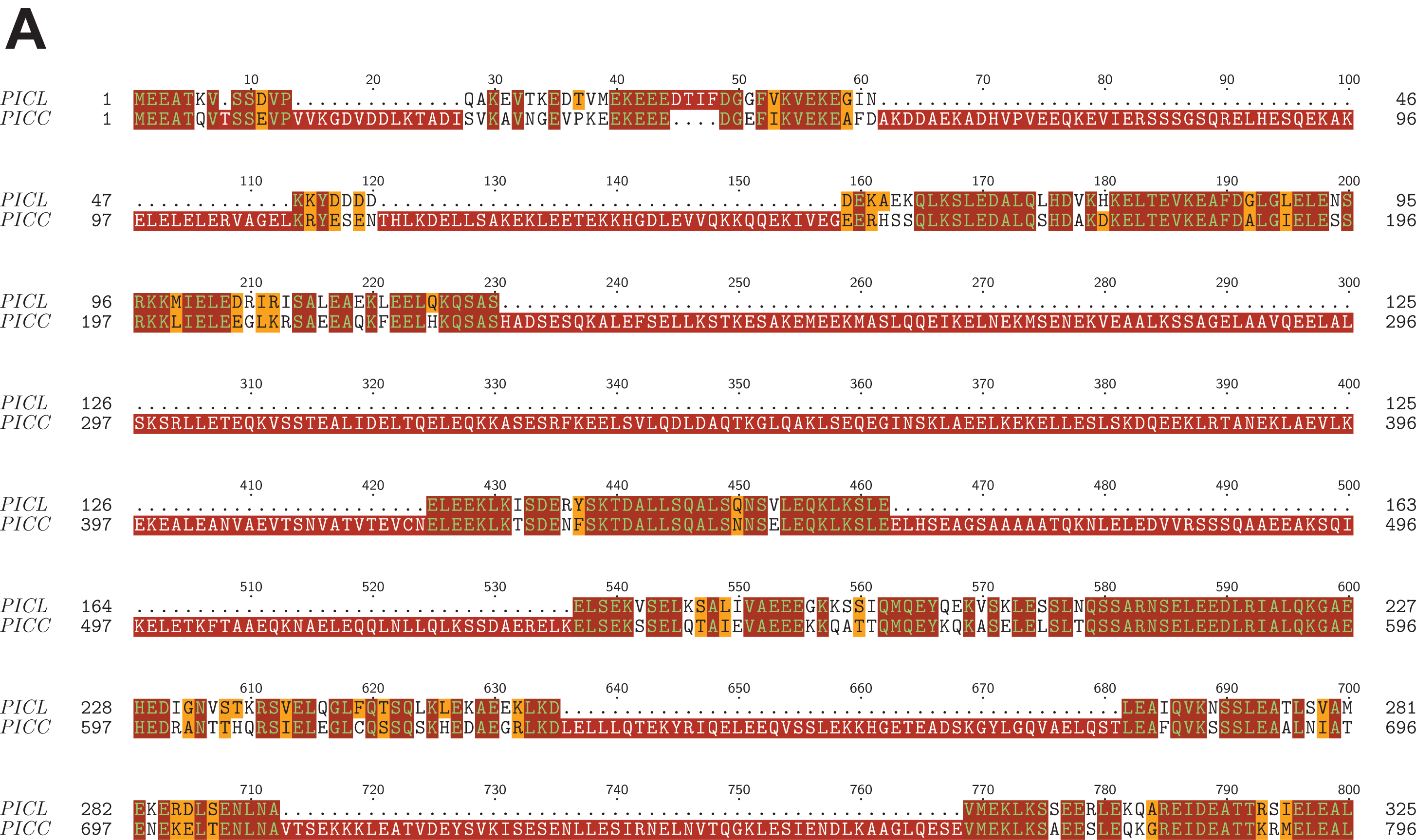


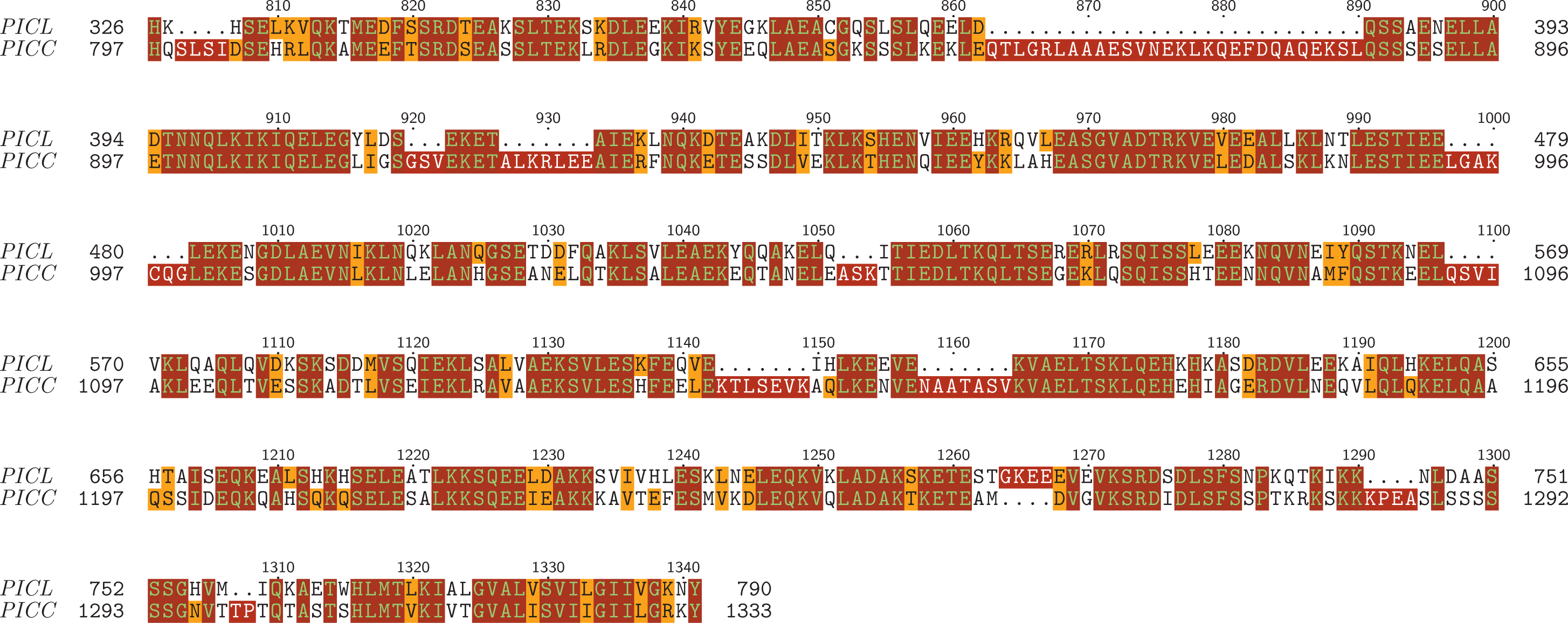


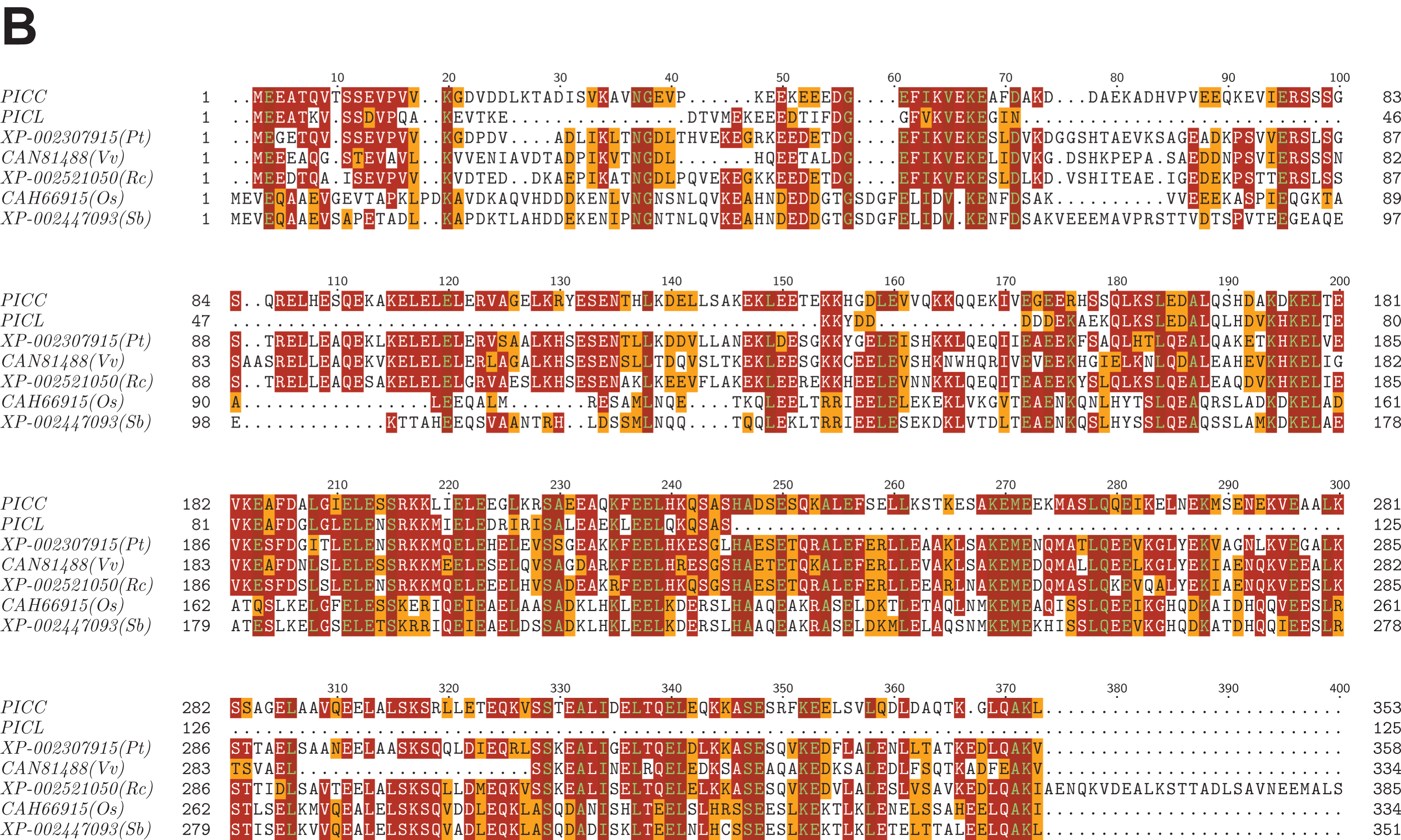


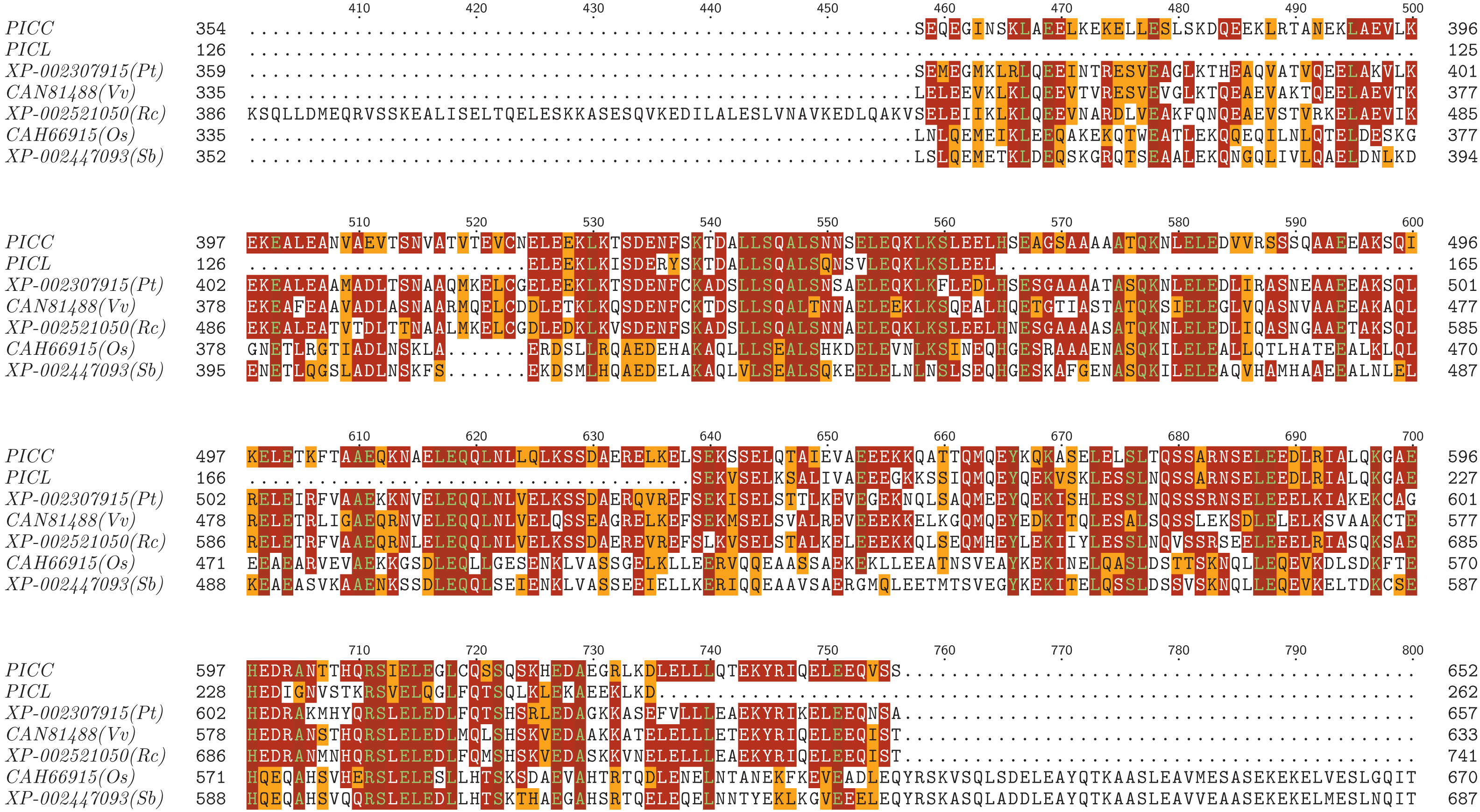


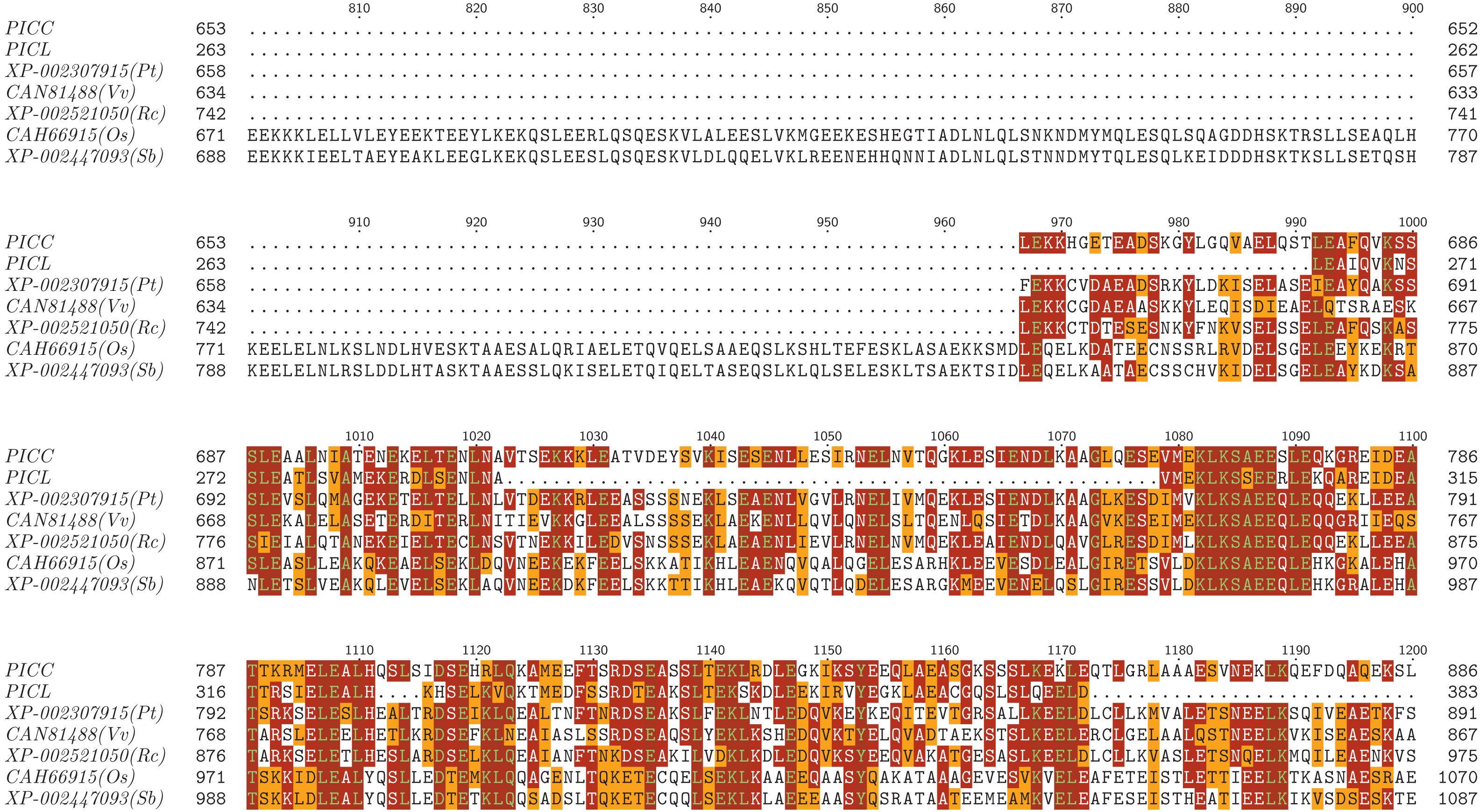


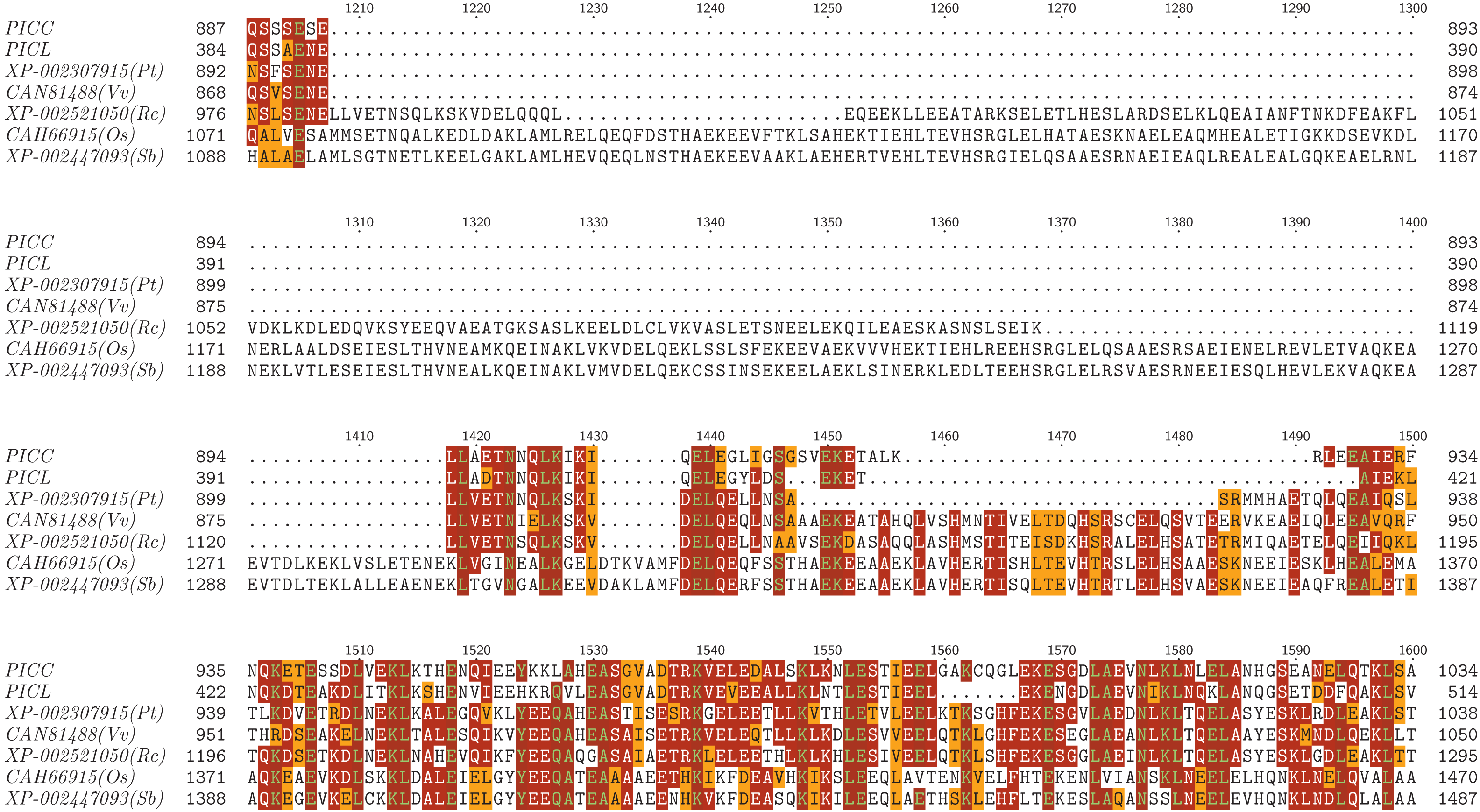


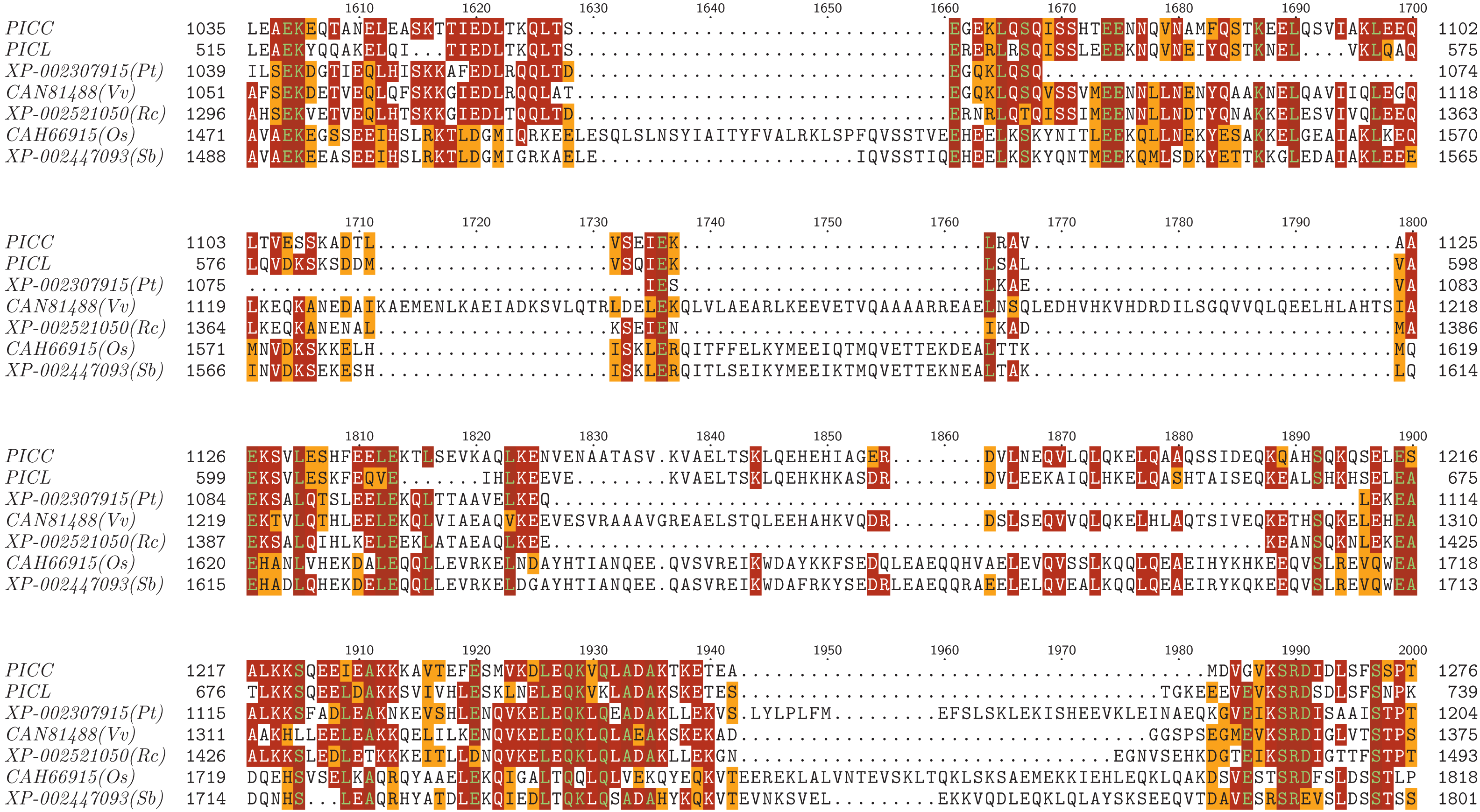


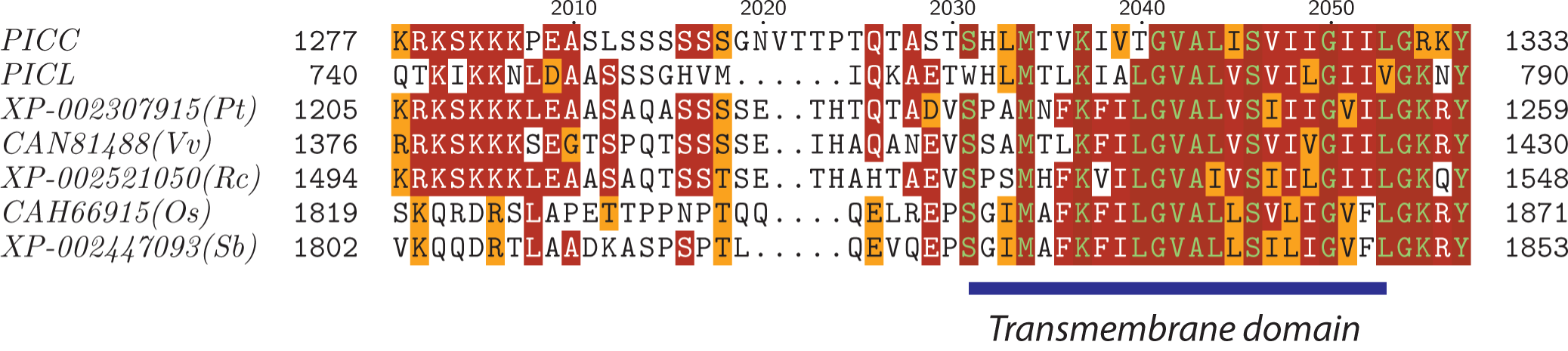


**Figure S1. (A)** Sequence alignment of PICC and PICL. **(B)** Multiple sequence alignment of PICC, PICL and their orthologs in vascular plants. Blue bar below the alignment indicates the predicted transmembrane domain. Os, *Oryza sativa*; Pt, *Populus trichocarpa;* Rc, *Ricinus communis;* Sb*, Sorghum bicolor;* Vv, *Vitis vinifera.*
