# Supplementary material for: Functional Investigation of the Plant-Specific Long Coiled-Coil Proteins PAMP-INDUCED COILED-COIL (PICC) and PICC-LIKE (PICL) in Arabidopsis thaliana
Source: PLoS One. 2013 Feb 25;8(2):e57283. doi: 10.1371/journal.pone.0057283 (PMC3581476; doi:10.1371/journal.pone.0057283)
Supplement: Figure S2 — Expression pattern of PICC and PICL in various organs. PICC (At2g32240) and PICL (At1g05320) expression pattern based on microarray expression data using the AtGenExpress Visualization Tool [54]. Each point indicates an expression value from an independent experiment [54]. (DOCX) [file pone.0057283.s002.docx]

**

**

**Figure S2. Expression pattern of *PICC* and *PICL* in various organs***. PICC* (At2g32240) and *PICL* (At1g05320) expression pattern based on microarray expression data using the AtGenExpress Visualization Tool [[1](#_ENREF_1)]. Each point indicates an expression value from an independent experiment [[1](#_ENREF_1)].

**SUPPorting LITERATURE CITED**

1. Schmid M, Davison TS, Henz SR, Pape UJ, Demar M, et al. (2005) A gene expression map of Arabidopsis thaliana development. Nat Genet 37: 501-506.
