# Supplementary material for: Functional Investigation of the Plant-Specific Long Coiled-Coil Proteins PAMP-INDUCED COILED-COIL (PICC) and PICC-LIKE (PICL) in Arabidopsis thaliana
Source: PLoS One. 2013 Feb 25;8(2):e57283. doi: 10.1371/journal.pone.0057283 (PMC3581476; doi:10.1371/journal.pone.0057283)
Supplement: Figure S3 — T-DNA insertion alleles of PICL and PICC. (A) Genomic structure of PICL and PICC showing T-DNA insertion sites in picl-1, picc-1 and picc-2 alleles. Black asterisk (*) indicates the region encoding the antigen (amino acids 1–100) used for anti-PICC/PICL antibody development. This sequence is highly conserved in PICC and PICL. (B) Immunoblot analysis using PICL antibody detecting the presence of full-length PICC and PICL and truncated PICL (tr.PICL) and truncated PICC (tr.PICC) in WT and mutant Arabidopsis protein extracts. Molecular mass markers are indicated on the right. Ponceau membrane stained with Ponceau S before immunoblotting, indicating close-to equal loading. The 50 kDa RBCS band is shown. (C) Total (T), microsomal (M) and soluble (S) fractions of WT and picl-1 Arabidopsis leaf protein extracts detected in an immunoblot with the PICL antibody. In WT, PICL is associated with the membrane and is detected in the microsomal fraction. In picl-1, the truncated protein lacks the transmembrane domain and is no longer associated with the membrane, which is evident by the absence of truncated PICL in the microsomal fraction. Ponceau-stained membrane is shown for loading control. (DOCX) [file pone.0057283.s003.docx]

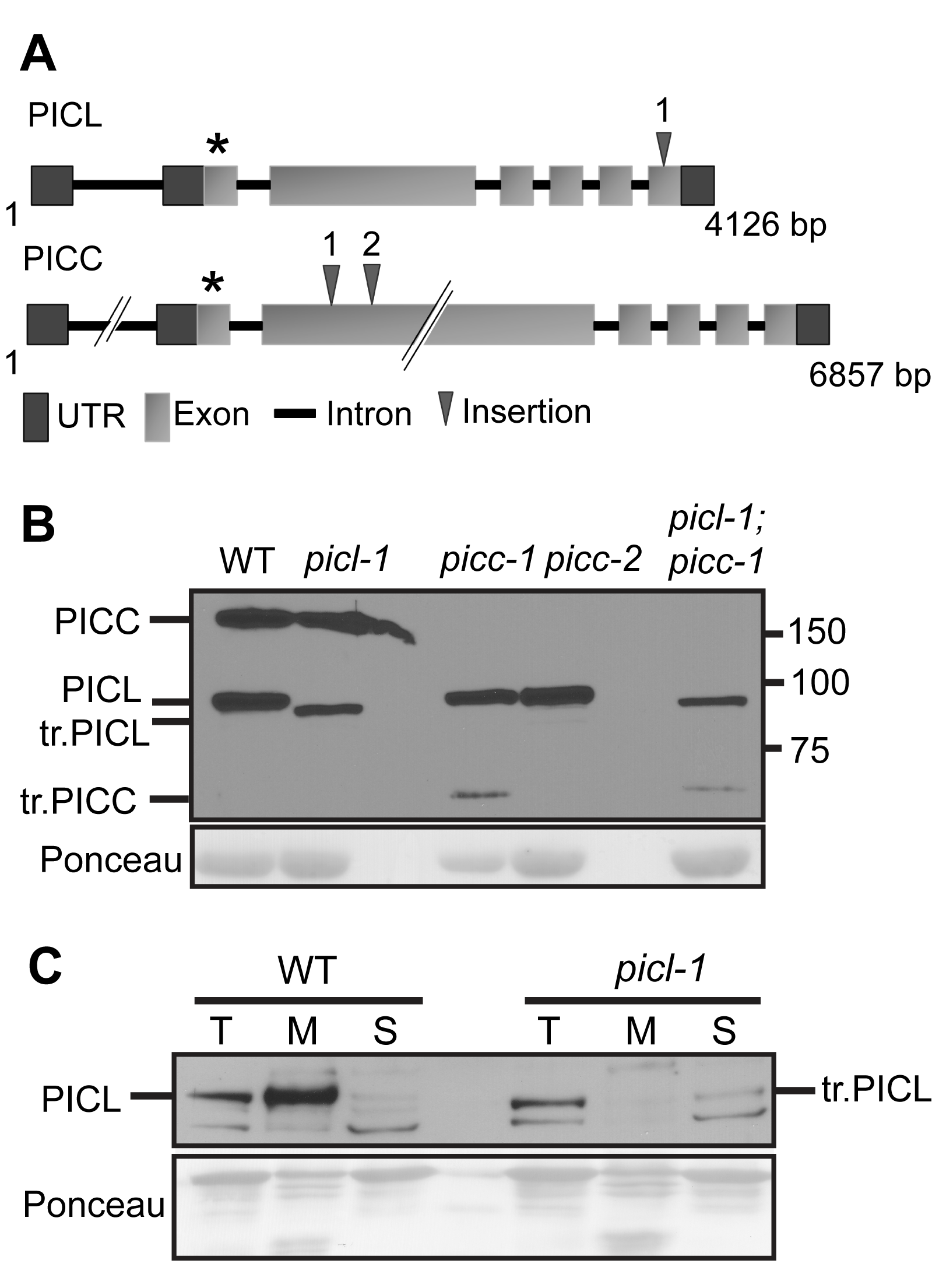


**Figure S3. T-DNA insertion alleles of PICL and PICC. (A)** Genomic structure of PICL and PICC showing T-DNA insertion sites in *picl-1*, *picc-1* and *picc-2* alleles. Black asterisk (*) indicates the region encoding the antigen (amino acids 1-100) used for anti-PICC/PICL antibody development. This sequence is highly conserved in PICC and PICL. **(B)** Immunoblot analysis using PICL antibody detecting the presence of full-length PICC and PICL and truncated PICL (tr.PICL) and truncated PICC (tr.PICC) in WT and mutant Arabidopsis protein extracts. Molecular mass markers are indicated on the right. Ponceau membrane stained with Ponceau S before immunoblotting, indicating close-to equal loading. The 50 kDa RBCS band is shown. **(C)** Total (T), microsomal (M) and soluble (S) fractions of WT and *picl-1* Arabidopsis leaf protein extracts detected in an immunoblot with the PICL antibody. In WT, PICL is associated with the membrane and is detected in the microsomal fraction. In *picl-1,* the truncated protein lacks the transmembrane domain and is no longer associated with the membrane, which is evident by the absence of truncated PICL in the microsomal fraction. Ponceau-stained membrane is shown for loading control.
