# Supplementary material for: Functional Investigation of the Plant-Specific Long Coiled-Coil Proteins PAMP-INDUCED COILED-COIL (PICC) and PICC-LIKE (PICL) in Arabidopsis thaliana
Source: PLoS One. 2013 Feb 25;8(2):e57283. doi: 10.1371/journal.pone.0057283 (PMC3581476; doi:10.1371/journal.pone.0057283)
Supplement: Figure S4 — ER morphology is not visibly altered in picc-1;picl-1 mutant plants. Confocal images of Arabidopsis leaves expressing HDEL-mCherry in WT (A and C) and picc-1;picl-1 (B and D). Scale = 10 µm. (DOCX) [file pone.0057283.s004.docx]

**
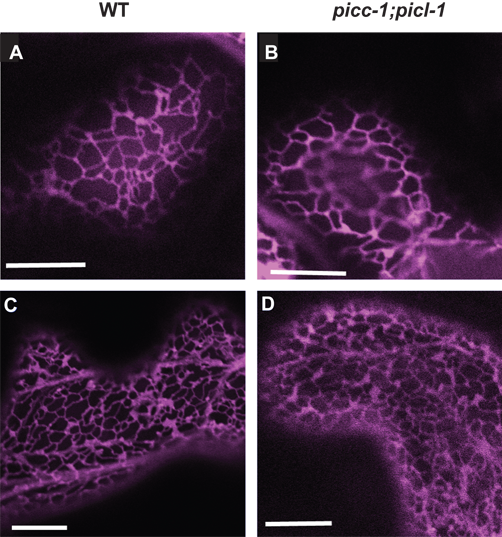
**

**Figure S4. ER morphology is not visibly altered in *picc-1;picl-1* mutant plants.** Confocal images of Arabidopsis leaves expressing HDEL-mCherry in WT (A and C) and *picc-1;picl-1* (B and D). Scale = 10 µm.
