# Supplementary material for: Functional Investigation of the Plant-Specific Long Coiled-Coil Proteins PAMP-INDUCED COILED-COIL (PICC) and PICC-LIKE (PICL) in Arabidopsis thaliana
Source: PLoS One. 2013 Feb 25;8(2):e57283. doi: 10.1371/journal.pone.0057283 (PMC3581476; doi:10.1371/journal.pone.0057283)
Supplement: Figure S5 — picc-1, picc-2 and picc-1;picl-1 Arabidopsis plants are more susceptible to the avirulent bacterial strain hrcC. Levels of hrcC and PstDC3000 four days after infiltration into leaves of the indicated plants. Values represent average of three replicates. Error bars represent one standard deviation. CFU, Colony Forming Units. (DOCX) [file pone.0057283.s005.docx]

**
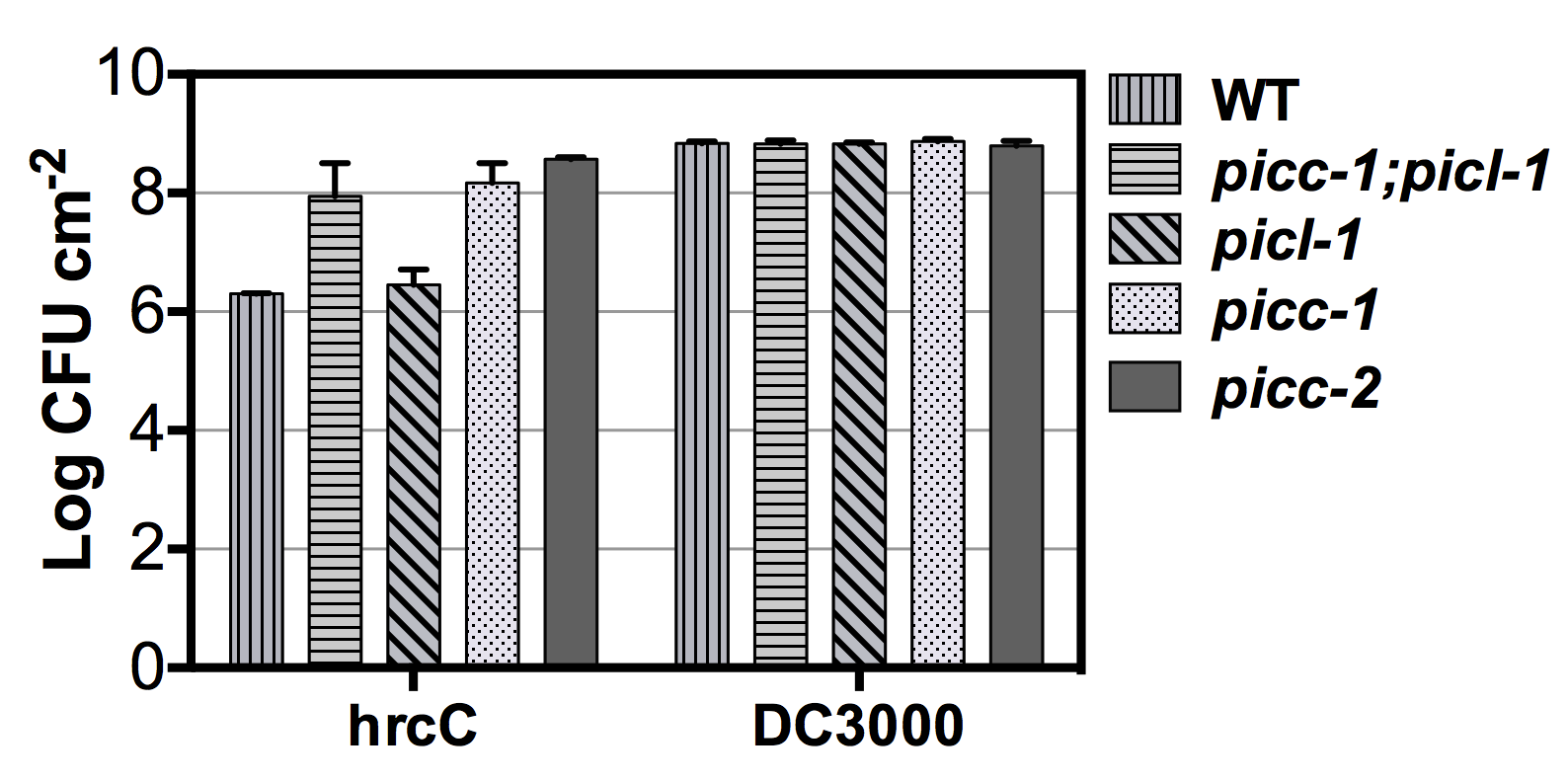
**

**Figure S5. *picc-1, picc-2* and *picc-1;picl-1* Arabidopsis plants are more susceptible to the avirulent bacterial strain *hrcC.*** Levels of *hrcC* and *Pst*DC3000 four days after infiltration into leaves of the indicated plants. Values represent average of three replicates. Error bars represent one standard deviation. CFU, Colony Forming Units.
