# Supplementary material for: Functional Investigation of the Plant-Specific Long Coiled-Coil Proteins PAMP-INDUCED COILED-COIL (PICC) and PICC-LIKE (PICL) in Arabidopsis thaliana
Source: PLoS One. 2013 Feb 25;8(2):e57283. doi: 10.1371/journal.pone.0057283 (PMC3581476; doi:10.1371/journal.pone.0057283)
Supplement: Figure S6 — flg22-induced resistance against Pst DC3000 is not compromised in picl-1 , picc-1 or picc-1;picl-1 mutant plants. Bacterial suspension of PstDC3000 was infiltrated into indicated plants 24 h after pretreatment with water (mock) or 1 µM flg22 (flg22). Values represent average of three replicates. Error bars represent one standard deviation. Similar results were obtained in two biological replicates. CFU, Colony Forming Units. (DOCX) [file pone.0057283.s006.docx]

**
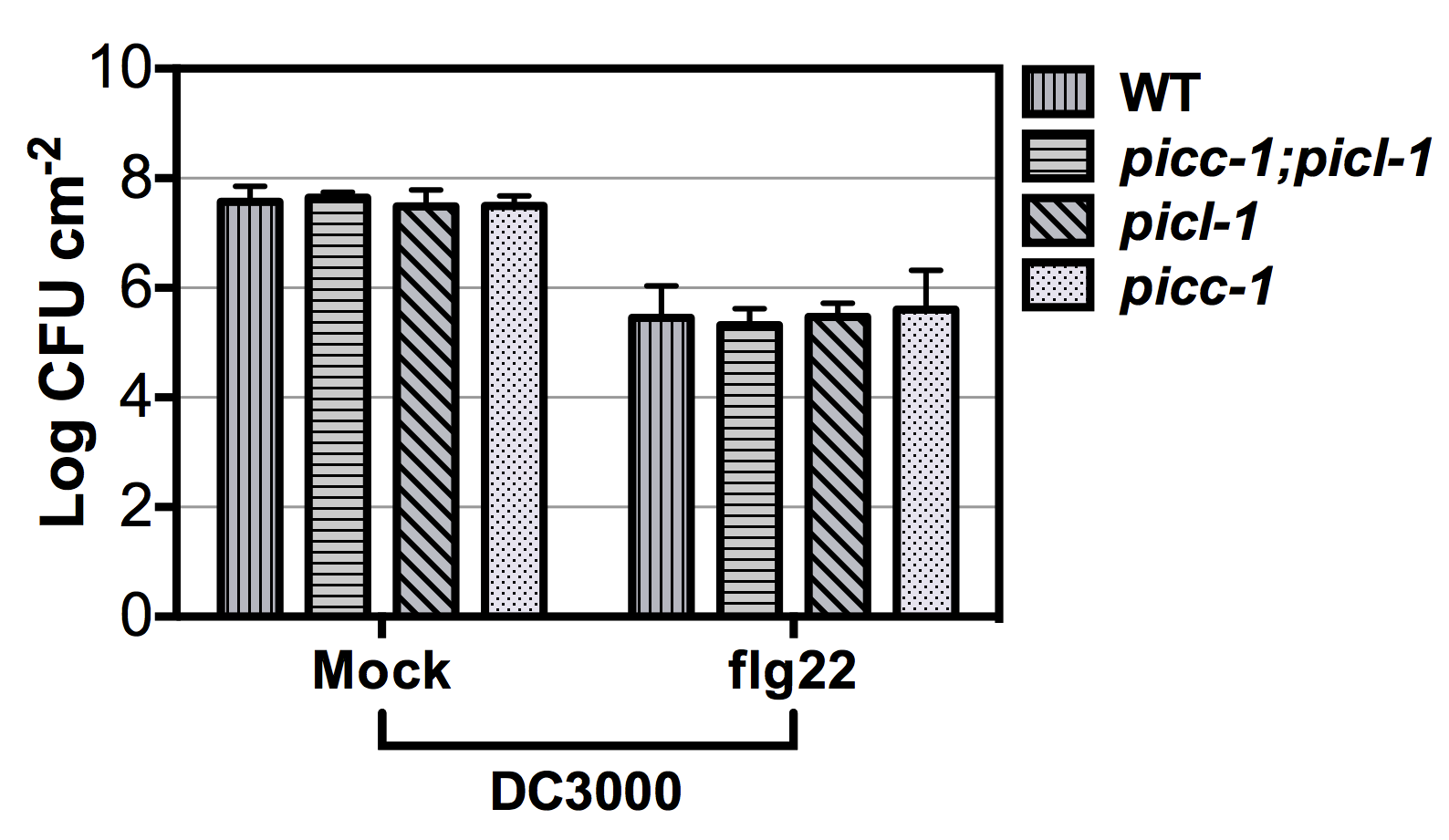
**

**Figure S6. flg22-induced resistance against *Pst*DC3000 is not compromised in *picl-1*, *picc-1* or *picc-1;picl-1* mutant plants.** Bacterial suspension of *Pst*DC3000 was infiltrated into indicated plants 24 h after pretreatment with water (mock) or 1 μM flg22 (flg22). Values represent average of three replicates. Error bars represent one standard deviation. Similar results were obtained in two biological replicates. CFU, Colony Forming Units.
