# Supplementary material for: Functional Investigation of the Plant-Specific Long Coiled-Coil Proteins PAMP-INDUCED COILED-COIL (PICC) and PICC-LIKE (PICL) in Arabidopsis thaliana
Source: PLoS One. 2013 Feb 25;8(2):e57283. doi: 10.1371/journal.pone.0057283 (PMC3581476; doi:10.1371/journal.pone.0057283)
Supplement: Figure S7 — Generation of reactive oxygen species is not compromised in picc-1 . (A) Total ROS generation triggered by 10 µM flg22 in WT and picc-1 represented as a percentage of WT. Values represent average of three biological replicates. Error bars represent one standard deviation. (B) A time trace of the flg22 triggered oxidative outburst in WT and picc-1. WT and picc-1 treated with mock (water) are shown as negative controls. Similar results were obtained in three biological replicates. (DOCX) [file pone.0057283.s007.docx]

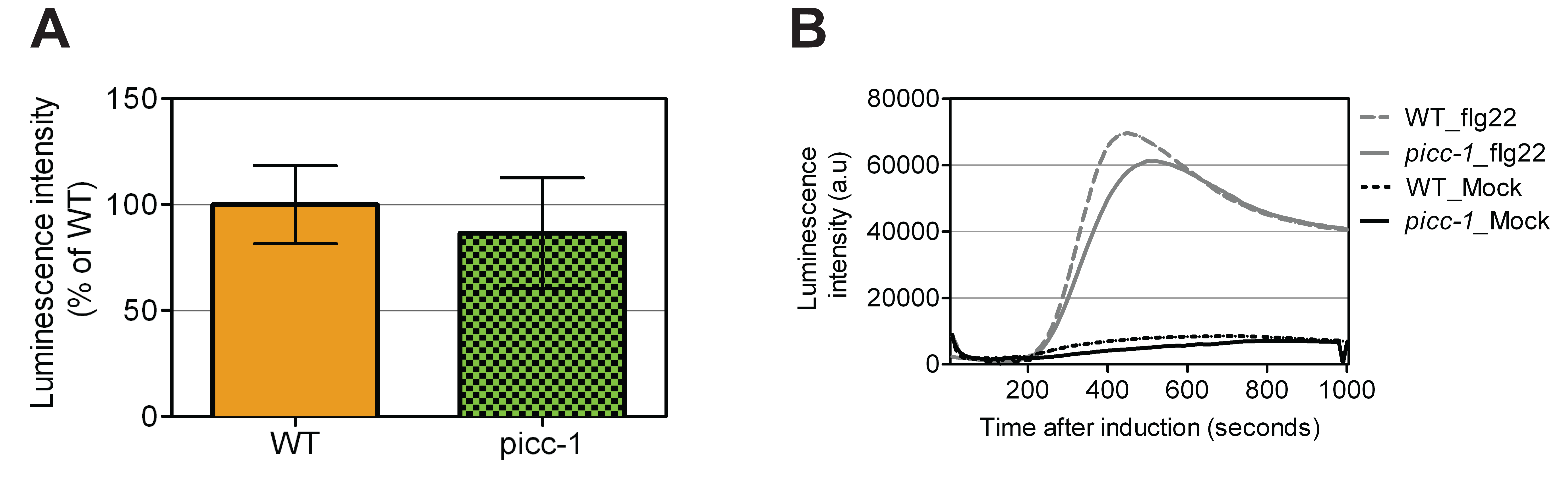


**Figure S7. Generation of reactive oxygen species is not compromised in *picc-1*. (A)** Total ROS generation triggered by 10 μM flg22 in WT and *picc-1* represented as a percentage of WT. Values represent average of three biological replicates. Error bars represent one standard deviation. **(B)** A time trace of the flg22 triggered oxidative outburst in WT and *picc-1*. WT and *picc-1* treated with mock (water) are shown as negative controls. Similar results were obtained in three biological replicates.
