# Supplementary material for: Functional Investigation of the Plant-Specific Long Coiled-Coil Proteins PAMP-INDUCED COILED-COIL (PICC) and PICC-LIKE (PICL) in Arabidopsis thaliana
Source: PLoS One. 2013 Feb 25;8(2):e57283. doi: 10.1371/journal.pone.0057283 (PMC3581476; doi:10.1371/journal.pone.0057283)
Supplement: Figure S8 — PAMP-induced expression changes are not altered in picc-1. Leaves from 4-week-old WT and picc-1 plants were infiltrated with 1 µM flg22 or bacterial suspensions (2×10^8^ CFU ml−1) of type III secretion deficient hrcC. Steady state mRNA levels of (A) MYB51, (B) ICS1, (C) PR1 and (D) NCED3 were quantified by real-time PCR at times indicated. Transcript levels were normalized to ACTIN levels from the same sample. Values are given in arbitrary units with the value in 24 h WT samples infiltrated with either flg22 or hrcC set to 1. Each value is represented as an average of three biological replicates. Error bars indicate one standard deviation. (DOCX) [file pone.0057283.s008.docx]

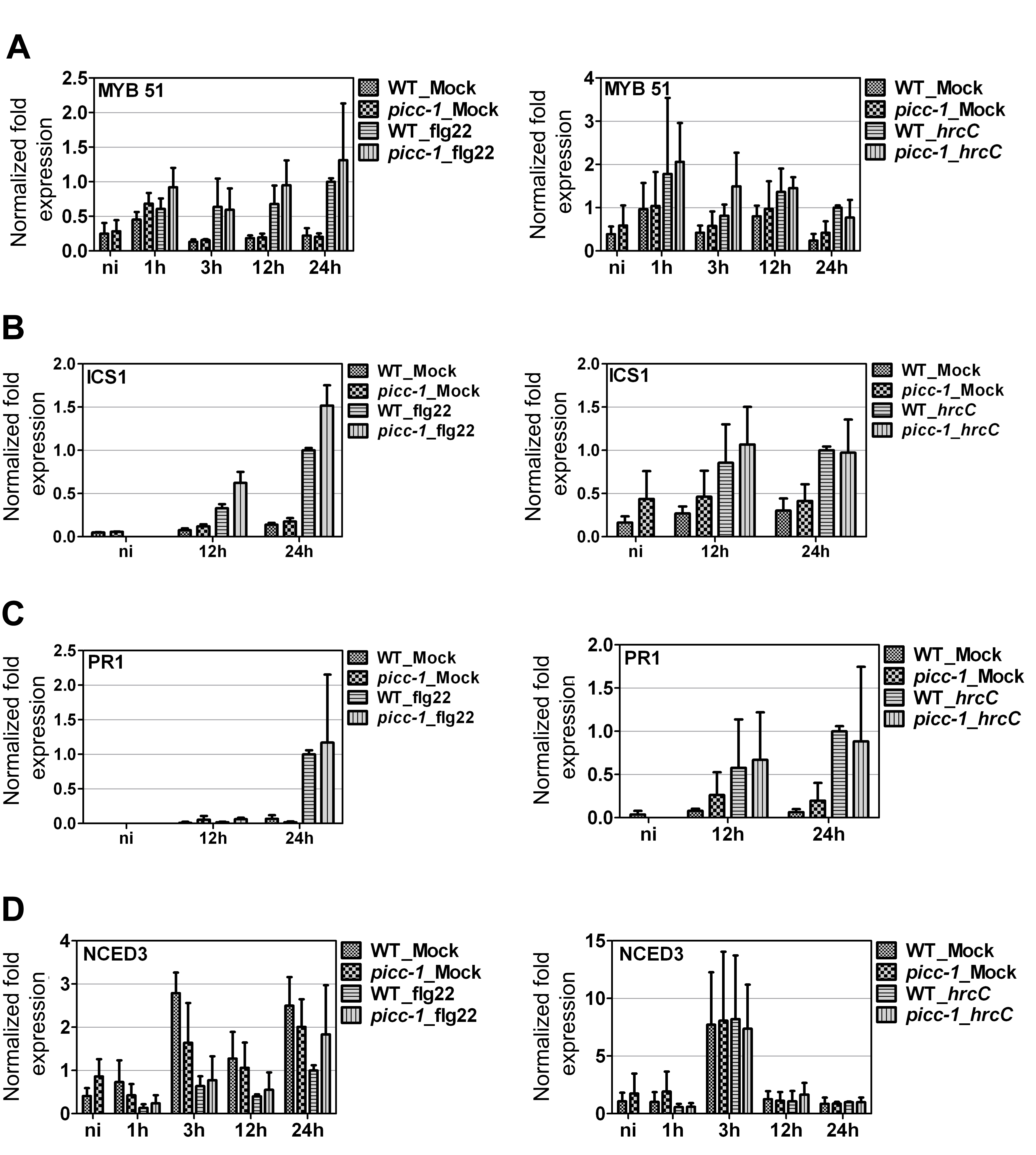


**Figure S8. PAMP-induced expression changes are not altered in *picc-1.*** Leaves from 4-week-old WT and *picc-1* plants were infiltrated with 1 μM flg22 or bacterial suspensions (2 x 10^8^ CFU ml^-1^) of type III secretion deficient *hrcC*. Steady state mRNA levels of **(A)** *MYB51*, **(B)** *ICS1*, **(C)** *PR1* and **(D)** *NCED3* were quantified by real-time PCR at times indicated. Transcript levels were normalized to *ACTIN* levels from the same sample. Values are given in arbitrary units with the value in 24 h WT samples infiltrated with either flg22 or *hrcC* set to 1. Each value is represented as an average of three biological replicates. Error bars indicate one standard deviation.
