# Supplementary material for: Functional Investigation of the Plant-Specific Long Coiled-Coil Proteins PAMP-INDUCED COILED-COIL (PICC) and PICC-LIKE (PICL) in Arabidopsis thaliana
Source: PLoS One. 2013 Feb 25;8(2):e57283. doi: 10.1371/journal.pone.0057283 (PMC3581476; doi:10.1371/journal.pone.0057283)
Supplement: Table S1 — Primers used for genotyping. (DOCX) [file pone.0057283.s009.docx]

| **Primer name** | **Primer sequence (5’ – 3’)** | **Source** |
| --- | --- | --- |
| PICC_58801_LP | GCTTGCGGAAGAACTCAAGGAG | This study |
| PICC_58801_RP | CTTCTTCGGCAACTTCAATAGCAG | This study |
| PICC_139837_LP | CAGTATTGAACTAGAAGG | This study |
| PICC_139837_RP | CTGGTAGTGAACTCCTCCATTG | This study |
| PICL_56040_LP | gcttgcagatgctaaatcttaag | This study |
| PICL_56040_RP | tatggtagaagcaaaaattcttaga | This study |
| Lba1 | TGGTTCACGTAGTGGGCCATCG | http://signal.salk.edu /tdnaprimers.2.html |

Table S1. Primers used for genotyping.
