# Supplementary material for: Functional Investigation of the Plant-Specific Long Coiled-Coil Proteins PAMP-INDUCED COILED-COIL (PICC) and PICC-LIKE (PICL) in Arabidopsis thaliana
Source: PLoS One. 2013 Feb 25;8(2):e57283. doi: 10.1371/journal.pone.0057283 (PMC3581476; doi:10.1371/journal.pone.0057283)
Supplement: Table S2 — Primers used for cloning. (DOCX) [file pone.0057283.s010.docx]

| **Primer Name** | **Primer sequence (5’ – 3’)** | **Source** |
| --- | --- | --- |
| PICCattB1F | GGGGACAAGTTTGTACAAAAAAGCAGGCTTCATGGAAGAAGCAACTCAAGTAACG | This study |
| PICCattB2R | GGGGACCACTTTGTACAAGAAAGCTGGGTCCTAATACTTTCTCCCAAGAATTATACC | This study |
| PICLpentrF | CACCATGGAAGAAGCAACAAAA | This study |
| PICLpentrR | TCAATAATTTTTCCCAACAATGAT | This study |
| PICCΔTDFattB1F | GGGGACAAGTTTGTACAAAAAAGCAGGCTTCATGGAAGAAGCAACTCAAGTAACG | This study |
| PICCΔTDFattB2R | GGGGACCACTTTGTACAAGAAAGCTGGGTCctatgtttgagtaggagtagtaac | This study |
| PICLΔTDFattB1F | GGGGACAAGTTTGTACAAAAAAGCAGGCTTCATGGAAGAAGCAACAAAA | This study |
| PICLΔTDFattB2R | GGGGACCACTTTGTACAAGAAAGCTGGGTCctactgaatcataacgtgccctg | This study |
| TDF^PICC^attB1F | GGGGACAAGTTTGTACAAAAAAGCAGGCTTCGCATCAACTTCACATCTCATGACAG | This study |
| TDF^PICC^attB2R | GGGGACCACTTTGTACAAGAAAGCTGGGTCCTAATACTTTCTCCCAAGAATTATACC | This study |
| TDF^PICL^attB1F | GGGGACAAGTTTGTACAAAAAAGCAGGCTTCAAAGCTGAAACATGGCATCTCATG | This study |
| TDF^PICL^attB2R | GGGGACCACTTTGTACAAGAAAGCTGGGTCTCAATAATTTTTCCCAACAATGATAC | This study |
| prPICCattB1F | GGGGACAAGTTTGTACAAAAAAGCAGGCTCGGTGTTGTGAACGGATTTAGAAGG | This study |
| prPICCattB2R | GGGGACCACTTTGTACAAGAAAGCTGGGTCCTGAGTGTTCGCCTGTTTTTTCTCTTC | This study |
| PICCpBT3NF | GAATTCCTGCAGGGCCATTACGGCCATGGAAGAAGCAACTCAAGTAAC | This study |

| PICCpBT3NR | CTACTTACCATGGGGCCGAGGCGGCCTTTTAATACTTTCTCCCAAGAATTATAC | This study |
| --- | --- | --- |
| PICLpBT3NF | GAATTCCTGCAGGGCCATTACGGCCATGGAAGAAGCAACAAAAGTGAG | This study |
| PICLpBT3NR | CTACTTACCATGGGGCCGAGGCGGCCTTTTAATAATTTTTCCCAACAATGATAC | This study |
| PICCpPR3NF | CAACGCAGAGTGGCCATTACGGCCATGGAAGAAGCAACTCAAGTAAC | This study |
| PICCpPR3NR | GAATTCTCGAGAGGCCGAGGCGGCCTTAATACTTTCTCCCAAGAATTATAC | This study |
| PICLpPR3NF | CAACGCAGAGTGGCCATTACGGCCATGGAAGAAGCAACAAAAGTGAG | This study |
| PICLpPR3NR | GAATTCTCGAGAGGCCGAGGCGGCCTTAATAATTTTTCCCAACAATGATAC | This study |

Table S2. Primers used for cloning.
