# Supplementary material for: Functional Investigation of the Plant-Specific Long Coiled-Coil Proteins PAMP-INDUCED COILED-COIL (PICC) and PICC-LIKE (PICL) in Arabidopsis thaliana
Source: PLoS One. 2013 Feb 25;8(2):e57283. doi: 10.1371/journal.pone.0057283 (PMC3581476; doi:10.1371/journal.pone.0057283)
Supplement: Table S3 — Primers used for real-time PCR. (DOCX) [file pone.0057283.s011.docx]

| **Primer Name** | **Primer Sequence (5’ – 3’)** | **Source** |
| --- | --- | --- |
| PICC_F | CGAGAAGGAGCAAACAGCCAATG | This study |
| PICC_R | CCTCAGTGTGGGAAGAAATCTGTG | This study |
| PICL_F | cattcgattcagcaccttcaacgg | This study |
| PICL_R | CCTTAGCTTGAGGGACGTCTGAAC | This study |
| MYB51_F | CCTTCACGGCAACAAATGGTCTG | This study |
| MYB51_R | TACCGGAGGTTATGCCCTTGTG | This study |
| ICS1_F | GCTTGGCTAGCACAGTTACAGC | This study |
| ICS1_R | CACTGCAGACACCTAATTGAGTCC | This study |
| PR1_F | CTACGCAGAACAACTAAGAGGCAAC | This study |
| PR1_R | TTGGCACATCCGAGTCTCACTG | This study |
| NCED3_F | AGCTCCTTACCTATGGCCAGT | [[1](#_ENREF_1)] |
| NCED3_R | CGCTCTCTGGAACAAATTCATC | [[1](#_ENREF_1)] |
| Actin_F | CTAAGCTCTCAAGATCAAAGGCTTA | [[2](#_ENREF_2)] |
| Actin_R | TTAACATTGCAAAGAGTTTCAAGGT | [[2](#_ENREF_2)] |

Table S3. Primers used for real-time PCR.

**SUPPORTING LITERATURE CITED**

1. De Torres Zabala M, Bennett MH, Truman WH, Grant MR (2009) Antagonism between salicylic and abscisic acid reflects early host–pathogen conflict and moulds plant defence responses. The Plant Journal 59: 375-386.

2. An Y-Q, McDowell JM, Huang S, McKinney EC, Chambliss S, et al. (1996) Strong, constitutive expression of the Arabidopsis ACT2/ACT8 actin subclass in vegetative tissues. The Plant Journal 10: 107-121.
